# Visual and Verbal Working Memory and Processing Speed Across the Adult Lifespan: The Effect of Age, Sex, Educational Level, Awakeness, and Hearing Sensitivity

**DOI:** 10.3389/fpsyg.2021.668828

**Published:** 2021-10-14

**Authors:** Katrien Kestens, Sofie Degeest, Marijke Miatton, Hannah Keppler

**Affiliations:** ^1^Department of Rehabilitation Sciences, Ghent University, Ghent, Belgium; ^2^Department of Head and Skin, Ghent University Hospital, Ghent University, Ghent, Belgium; ^3^Department of Oto-Rhino-Laryngology, Ghent University Hospital, Ghent, Belgium

**Keywords:** working memory, processing speed, cognitive construct, audiology, hearing loss

## Abstract

**Objective:** To strengthen future methodological choices regarding the measurement of cognition within the field of audiology, the current study aimed to examine the effect of, among other things, hearing sensitivity on the backward corsi tapping task (i.e., visual working memory and processing speed) and the letter-number sequencing task (i.e., verbal working memory and processing speed).

**Design and Study Sample:** The backward corsi tapping task and the letter-number sequencing task were administered to 184 participants, aged between 18 and 69 years. The effect of age, sex, educational level, awakeness, and hearing sensitivity on verbal and visual working memory and processing speed was assessed using stepwise multiple regression analyses.

**Results:** For all outcome variables, a decrease in performance was observed with increasing age. For visual and verbal working memory, males outperformed females, whereas no clear sex effect was observed for visual and verbal processing speed. Hearing sensitivity had only a significant impact on visual processing speed.

**Conclusion:** The importance to evaluate cognitive construct validity within audiological research was highlighted. Further research should focus on investigating the associations between speech understanding on the one hand and the backward corsi tapping task and letter-number sequencing task on the other hand.

## Introduction

Hearing can be described as a passive function that requires bottom-up processes in order to detect, localize, and discriminate sounds (Kiessling et al., 2003). Speech understanding, however, is the active process of hearing, requiring both bottom-up and top-down processes. In addition to the peripheral and central auditory system, cognitive resources are utilized to understand speech, especially in complex listening conditions (Rönnberg et al., 2013; Pichora-Fuller et al., 2016).

Working memory (i.e., limited capacity system, which temporarily stores and process information) and processing speed (i.e., rate at which cognitive processing occurs) were reported as the most important predictors for speech understanding, especially among hearing-impaired elderly (Vaughan et al., 2006; Dryden et al., 2017). Their contribution during speech understanding has been described within the Ease of Language Understanding (ELU) model (Rönnberg et al., 2013). Within this model, the match between the mental representation of the incoming speech signal and its storage in the semantic long-term memory determines the cognitive effort to understand speech. Distorsions of the incoming speech signal, both internal (e.g., hearing loss) and external (e.g., environmental background noise), might hinder this matching process resulting in a so called mismatch. The greater the mismatch, the more explicit and slower cognitive processes are needed to resolve a match and to eventually understand the incoming speech signal. Specifically, working memory is used to retain earlier parts of the incoming speech signal until they can be integrated with the later parts. Processing speed plays a role during retrieval from, and comparison with semantic long-term memory. This expended attentional and cognitive effort used to understand speech is defined as listening effort (Hicks and Tharpe, 2002; Pichora-Fuller et al., 2016). Seemingly, this cognitive contribution during speech understanding might not be independently related to a listener’s age and hearing sensitivity (Füllgrabe and Rosen, 2016).

Recently, a more holistic approach of patient-centered care is given more attention in audiological research and practice (Meyer et al., 2016), which has, among other things, led to an increase in research within the field of cognitive hearing sciences (Kiessling et al., 2003; Kricos, 2006; Arlinger et al., 2009). In this respect, cognitive tests have been extensively used within audiological research, among other things, to unravel the auditory-cognitive perspective of speech understanding and listening effort (e.g., Akeroyd, 2008; Dryden et al., 2017; Harvey et al., 2017), to determine the relationship between cognitive decline and age-related hearing loss (e.g., Wayne and Johnsrude, 2015), to determine the effect of hearing aid usage on cognitive decline (e.g., Ohlenforst et al., 2017), and to determine the relationship between cognition and the benefit obtained with hearing aids (e.g., Kestens et al., 2021a).

The usage of cognitive tests within audiological research is well established, hence it is extremely striking that generally accepted guidelines regarding their usage are lacking. Based on a recent review (Kestens et al., 2021a), it was shown that significant more visual cognitive testing was performed compared to auditory cognitive testing within audiological research. However, the difference in construct validity between visual and auditory cognitive testing within a hearing-related context is important to consider. Construct validity is about ensuring that the method of measurement matches the concept that is intended to be measured (O’Leary-Kelly and Vokurka, 1998). Audiological rehabilitation aims to improve everyday speech communication. In this respect, examining cognition through visual testing is likely not serving the purpose due to a lack of construct validity (Shen et al., 2020). For example, working memory cannot be considered as an indivisible system, but as a cooperation of two temporary storage systems, namely the phonological loop for storage of phonetical information and the visuospatial sketchpad for storage of visual or spatial information (Baddeley, 2003). Consequently, visual and auditory, and in particular verbal cognitive testing differ in terms of the cognitive construct that is measured. The latter raises the question whether visual testing is sufficiently specific when conducted in a prominent hearing-related context. Indeed, verbal working memory emerged as the most salient predictor for speech understanding for older adults compared to its visual counterpart (Vaughan et al., 2008). This result highlights the importance of cognitive construct validity, and thus additional research on this topic is needed to eventually formulate hard evidence-based recommendation on the use of cognitive tests within audiological research.

Auditory cognitive testing might be preferred based on cognitive construct validity, though the results obtained from auditory cognitive tests might be influenced by the presence of hearing loss in a greater extent compared to results of visual cognitive tests. Specifically, the auditorily presented test items might be less heard within hearing-impaired individuals. Consequently, their response might not be entirely accurate which might be due to perceptual deficits rather than cognitive decline (Füllgrabe, 2020a). Moreover, as aforementioned, the perceptual processing of auditory stimuli within hearing-impaired individuals might allocate more explicit and slower cognitive processing which may lead to fewer available cognitive resources that can be used toward the execution of the cognitive task itself. Hence, it was suggested that in addition to lower cognitive abilities due to comorbidity with hearing loss, auditory cognitive testing in adults with hearing loss may also negatively impact cognitive outcomes (Shen et al., 2016; Füllgrabe, 2020b).

Therefore, the current study aimed to investigate the effect of age, sex, awakeness, educational level, and hearing sensitivity on visual and auditory working memory and processing speed. These two cognitive functions were chosen as they were reported as the most important predictors for speech understanding, especially among hearing-impaired elderly (Vaughan et al., 2006; Dryden et al., 2017). These results will strengthen future methodological choices regarding the measurement of cognition within the field of audiology and will eventually contribute in formulating evidence-based guidelines on how best measuring cognition within audiological practice.

## Materials and Methods

This study was approved by the local Ethics Committee and was conducted in accordance with the Helsinki Declaration. Participants provided their written informed consent to participate in this study.

### Participants

Flemish participants aged between 18 and 69 years with at least 12 years of education (i.e., high school graduate) were included in the study by means of convenience sampling. All participants had a normal middle ear status based on otoscopy and 226 Hz tympanometry (Amplivox Otowave 102 tympanometer). Air conduction pure-tone thresholds were bilaterally obtained for all octave frequencies between 0.125 and 8.00 kHz using the modified Hughson-Westlake method (Calisto audiometer, Interacoustics). Participants were included when air-conduction thresholds were symmetrical and equal to or better than the 95th percentile for age- and sex-adjusted thresholds norms [7029 International Organization for Standardization [ISO], 2017]. Asymmetric hearing was defined as a difference of 15 dB or more between the right and left ears at three contiguous frequencies. Pure-tone audiometry was conducted in a quiet room, assuring the ISO 8253-1 guidelines regarding the ambient sound pressure levels for accurate testing pure-tone thresholds [International Organization for Standardization (ISO), 2010]. Furthermore, it was ensured that all participants had normal or corrected-to-normal nearfield vision according to the Near Vision Snellen Eye Chart (Snellen, 1873). Participants with self-reported tinnitus, learning disorders, attention deficits, psychiatric disorders, or (a history of) neurological disorders were excluded. The Montréal Cognitive Assessment was carried out in participants aged 60 years or older, whereby participants who scored 25 or less were excluded to eliminate participants with a risk for cognitive impairment (Nasreddine et al., 2005). Last, participant’s awakeness was evaluated using a visual analog scale (0 = tired; 10 = totally awake). Participants indicating an awakeness score less than five were excluded (Kestens et al., 2021b) to rule out possible decreased cognitive functioning related to self-reported awakeness (Kronholm et al., 2009).

### Test Selection

The purpose of the authors was to make a clear distinction between tests in which the test items were presented visually and auditorily. An auditory, and in particular a verbal working memory task, i.e., the letter-number sequencing task, significantly predicted speech understanding in noise in older adults (Vaughan et al., 2008; Cox and Xu, 2010; Heinrich et al., 2019). Moreover, the letter-number sequencing task has been proven to be useful in the diagnosis of speech understanding deficits, aural rehabilitation, and training strategies (Vaughan et al., 2008). Another advantage of the letter-number sequencing task is that it is a standardized test, included in both the Wechsler Adult Intelligence scale (WAIS) and Wechsler Memory Scale (WMS). The letter-number sequencing task was recently added to these intelligence scales because it would tap more into working memory compared to the digit backward task. Although the latter was not confirmed by Egeland (2015), most studies indicated that the letter-number sequencing task relies primarily on verbal working memory (Crowe, 2000; Haut et al., 2000). Because of the above mentioned advantages, the letter-number sequencing task seemed a good candidate for use in audiological research (Vaughan et al., 2008) and was therefore chosen as the auditory, and in particular verbal working memory test (further denoted as verbal working memory).

Concerning visual working memory, the commonly used reading span test (Daneman and Carpenter, 1980) has been shown to be a good predictor of speech understanding in noise, especially in hearing-impaired elderly (Foo et al., 2007; Akeroyd, 2008). However, the reading span test is more considered a visual-verbal working memory test (Rönnberg et al., 2013) than a pure visual test, and thus, did not suit the current study purpose. Moreover, the reading span test requires a verbal response. As the used auditory test consisted of verbally presented test items, it seemed also important to not consider a verbal response within the visual test. Specifically, Baddeley’s working memory model stated that if a test would require recall of the visually presented stimuli, the visual input will, after visual analysis, be recoded verbally and thus, might access verbal cognitive pathways or resources (Baddeley, 2003). In this respect, a visual test without a verbal response was preferred. One of the most important visuo-spatial working memory tasks, used in neuropsychological research, is the backward corsi tapping task (Berch et al., 1998). To the best of our knowledge, the relationship between speech understanding (in noise) and performances on the backward corsi tapping task has not yet been investigated. Nevertheless, visuo-spatial working memory, measured with the backward corsi tapping task, has been shown to play an important role during speech-gesture integration processes (Wu and Coulson, 2014). Hence, the backward corsi tapping task might have significant applications in research regarding speech understanding in an auditory-visual context.

The letter-number sequencing task and the backward corsi tapping task are both standardized tests for measuring working memory, though their test design allow to simultaneously measure processing speed as well. In particular, the time needed to process the task can be measured and used as an outcome parameter of processing speed. As such, working memory and processing speed are examined in a time efficient manner.

### Test Procedure

All testing was performed by the same investigator (i.e., author KK) in a quiet room illuminated with standard room- and daylight. A randomization was used to determine the test sequence of the backward corsi tapping task and the letter-number sequencing task to rule out any order effect. The mean time for conducting each test was approximately 10 min.

#### Backward Corsi Tapping Task

A digital version of the existing corsi tapping task (WAIS-III-NL) was developed using the E-prime 2.0 software. The corsi raster (i.e., nine separated squares 30 mm × 30 mm large) was shown on a white 15.6 inch computer screen. Within this raster, series of identical blue filled circles appeared for 1 s each with an interstimulus interval of 1 s (see Supplementary Table 1 for an example). Participants had to memorize the squares in which circles appeared and had to indicate the position in reverse order (i.e., starting with the square were the last circle appeared and finishing with the square were the first circle appeared) by clicking the squares on the computer screen using a wireless computer mouse. It was obligated to click the correct amount of squares, even if guessing was necessary. Participants were instructed to accomplish this task as accurately and as quickly as possible. Once a response was given, self-corrections were not allowed. The span length increased successively from two to eight appearing circles. Two trials were given per sequence of the same span length. Two errors of the same span length resulted in termination of the task.

Participants were seated on a chair at eye height and in front of the computer screen at an individually determined distance in order to guarantee an optimal operation of the wireless computer mouse. At the beginning of the test, a practice trail was performed in order to verify participants’ computer skills and to verify if the squares could clearly be distinguished on the computer screen.

Three measures were determined to analyze visual working memory, namely span length (i.e., longest correctly recalled sequence; range: 2–8), raw score (i.e., number of correctly remembered trials; range: 0–14), and product score (i.e., compound score of span length and raw score; range: 0–112) (Kessels et al., 2008). To measure visual processing speed, the time clicking the last and first square was registered, using the E-prime 2.0 software. For each span length, the best performance of correctly solved trials was utilized in further analysis. The latter was done for several reasons. First, previous findings indicated significant differences in time-related outcomes between correctly and wrongly solved test items on the backward corsi tapping task (Brunetti et al., 2014). Second, the amount of correctly solved trials per span length might differ between participants. Hence, an average score of the correctly solved trials per span length was not preferred. Third, the worst score of the correctly solved items per span length could have been influenced by doubting or a moment of less concentration which might cause unwanted data points such as outliers.

#### Letter-Number Sequencing Task

The letter-number sequencing task (WAIS-IV-NL) contains of combinations of letters and numbers presented through live voice by a native Flemish speaker, at a normal speech rate (i.e., approximately 1.5 s per spoken stimulus) and normal loudness (i.e., approximately 65 dB SPL). Participants were instructed to recall the numbers in ascending order followed by the letters in alphabetical order as accurately and as quickly as possible. Once a response was given, self-corrections were not allowed. The span length successively increases from two up to eight. The amount of trials per sequence of the same span length varied between the presented span lengths. More specifically, for the sequences of a span length of two and three, two and three trials were administered, respectively. For all other span lengths (i.e., four up to eight) one trial was administered. Each trial consisted of three presented test items. Three errors of the same trial resulted in termination of the task.

Participants were seated on a chair in front of the examiner. At the beginning of the test, a practice trial was performed in order to verify whether the task was understood. If not, additional instructions were given until the participants fully understood the task.

Span length (range: 2–8), raw score (range: 0–30), and product score (range: 0–240) were determined to analyze verbal working memory (Kessels et al., 2008). Verbal processing speed was determined by measuring the time necessary to formulate an answer after the task was given by the investigator using a stopwatch for which well-defined guidelines were provided. The start button was pressed immediately after the task was given. The stop button was pressed at the end of a participant’s response. Specifically, the end of a response was determined when: (1) the correct sequence was given, (2) the total amount of letters and numbers were given, although incorrect, and (3) the participant indicated to have forgotten the sequence. Finally, the best item out of the correctly solved items per span length was included in further analysis.

### Statistical Analysis

Statistical analysis was conducted using SPSS version 24. A *p*-value of 0.05 was used as criterion of statistical significance. Descriptive parameters (i.e., mean, standard deviation, and range) were established for age, sex, awakeness, educational level, and hearing sensitivity. Educational level was counted as the amount of years a participant studied to obtain a specific educational degree, i.e., elementary school, high school, a professional or academic bachelor, a master’s degree, or a Ph.D. diploma. Full-time education was counted as full years; for part-time or evening education, the number of years was halved (e.g., 3 years of evening education was counted as 1.5 years). Hearing sensitivity was defined as the average of the thresholds at all measured frequencies of participants’ better ear.

For all variables examined during cognitive evaluation, descriptive analyses (i.e., mean, SD, range, percentile distribution, and percentage of correct responses) were conducted. For each cognitive outcome variable (i.e., dependent variables, *n* = 20), the most important predictors were selected by means of a stepwise multiple regression analysis. Within each stepwise multiple regression analysis age, sex, awakeness, educational level, and hearing sensitivity were subjected as the independent variables. The assumptions for an appropriate use of stepwise multiple regression analysis were checked (Laerd Statistics, 2015). First, independence of residuals was assessed by a Durbin–Watson statistic of approximately 2. Second, homoscedasticity of residuals was evaluated by using a scatterplot with the studentized residuals against the unstandardized predicted values. Third, multicollinearity was ruled out by inspecting the variance inflation factor (VIF) and correlation coefficients between independent variables which had to be equal or below 10 or 0.700, respectively. Fourth, unusual data points (i.e., outliers, high leverage points, and highly influential points) were identified. Significant outliers were identified when the standardized residual was greater than ± three SDs. To determine whether any cases exhibit high leverage, leverage values were considered and interpreted as safe (<0.20), risky (0.20–0.49), or dangerous ≥0.50 (Huber, 1981). Influential points were identified through a Cook’s Distance above 1. Last, the residuals needed to be approximately normally distributed which was graphically assessed though a histogram and Q-Q plot.

## Results

### Participants

In total, 199 participants took part of which 184 participants (85 males and 99 females) met all inclusion criteria. Table 1 shows detailed descriptions of age, sex, education level, awakeness, and hearing sensitivity per decade.

**TABLE 1:** Mean, standard deviation (SD), and range of age (years), educational level (years), awakeness, and hearing sensitivity (average of the thresholds at 0.125, 0.25, 0.50, 1.00, 2.00, 4.00, and 8.00 kHz of participants’ better ear, dB HL) per decade in total and for males, and females separately.

		Age (years)	Educational level (years)		Awakeness		Hearing sensitivity (dB HL)	
Decade	N	Mean (SD)	Mean (SD)	Range	Mean (SD)	Range	Mean (SD)	Range
18–29 years	Total (n = 58)	23.4 (3.1)	15.9 (1.9)	12.0–19.0	7.8 (1.2)	5.0–10.0	3.02 (3.63)	−7.86–13.57
	Male (n = 25)	23.9 (3.4)	15.8 (2.0)	12.0–19.0	8.2 (1.4)	5.0–10.0	2.71 (3.93)	−7.86–10.00
	Female (n = 33)	23.0 (2.9)	15.9 (1.7)	12.0–19.0	7.5 (0.9)	6.0–9.0	3.25 (3.44)	−2.86–13.57
30–39 years	Total (n = 31)	33.9 (2.8)	16.1 (3.5)	12.0–23.0	7.7 (1.4)	5.0–10.0	3.53 (3.14)	−1.43–13.57
	Male (n = 15)	34.0 (2.8)	14.8 (2.7)	12.0–21.0	7.4 (1.4)	5.0–10.0	4.24 (3.45)	0.71–13.57
	Female (n = 16)	33.7 (2.9)	17.4 (3.7)	12.0–23.0	8.0 (1.4)	5.0–10.0	2.86 (2.76)	−1.43–7.86
40–49 years	Total (n = 31)	44.2 (3.5)	15.0 (1.9)	12.0–19.0	7.9 (1.1)	6.0–10.0	4.42 (2.28)	0.71–27.14
	Male (n = 15)	42.5 (3.0)	15.3 (2.2)	12.0–19.0	7.8 (0.9)	6.0–9.0	4.38 (2.41)	0.71–8.57
	Female (n = 16)	45.8 (3.1)	14.9 (1.6)	12.0–17.0	8.0 (1.2)	6.0–10.0	4.46 (2.24)	1.43–8.57
50–59 years	Total (n = 34)	54.0 (2.6)	15.2 (1.9)	12.0–19.0	8.2 (0.9)	6.0–10.0	10.13 (5.49)	0.71–27.14
	Male (n = 15)	54.9 (2.6)	14.5 (2.0)	12.0–17.0	8.3 (0.7)	7.0–9.5	13.38 (5.79)	0.71–27.14
	Female (n = 19)	53.3 (2.4)	15.8 (1.6)	12.0–19.0	8.1 (1.1)	6.0–10.0	7.56 (3.67)	2.14–15.00
60–69 years	Total (n = 30)	63.9 (2.8)	14.5 (1.8)	12.0–18.0	8.6 (1.2)	5.0–10.0	12.98 (5.46)	2.86–24.29
	Male (n = 15)	63.8 (2.7)	14.9 (2.1)	12.0–18.0	8.8 (1.2)	5.0–10.0	11.90 (5.41)	3.57–23.57
	Female (n = 15)	64.1 (3.0)	14.0 (1.4)	12.0–16.0	8.4 (1.3)	5.0–10.0	14.05 (5.47)	2.86–24.29

### Working Memory

A detailed description (mean, SD, and range) of all outcome variables measuring visual and verbal working memory is shown in Tables 2, 3, respectively. Percentile distributions of these outcome variables are presented in digital Supplementary Tables 1, 2. Due to insufficient computer skills, one participant was excluded from the visual analysis as the obtained scores were considered not reliable. For both visual and verbal working memory, a ceiling effect was obtained for span length for all age decades, except for the oldest age group. Product score and raw score demonstrated more variance among outcome scores.

**TABLE 2:** Descriptive data for the backward corsi tapping task (i.e., visual working memory): span length (longest correctly remembered sequence, range: 2–8), raw score (number of correctly remembered trials, range: 0–14), and product score (compound score of span length and raw score, range: 0–112).

	Visual working memory						
		Span length		Raw score		Product score	
Decade	N	Mean (SD)	Range	Mean (SD)	Range	Mean (SD)	Range
All	Total (n = 183)	5.97 (1.30)	3.00–8.00	8.63 (2.31)	3.00–13.00	54.29 (23.98)	9.00–104.00
	Male (n = 85)	6.16 (1.17)	3.00–8.00	9.08 (2.24)	4.00–13.00	58.36 (23.68)	12.00–104.00
	Female (n = 98)	5.81 (1.38)	3.00–8.00	8.24 (2.31)	3.00–12.00	50.76 (23.80)	9.00–96.00
18–29	Total (n = 58)	6.62 (1.17)	4.00–8.00	9.72 (2.17)	4.00–13.00	66.52 (23.60)	16.00–104.00
	Male (n = 25)	6.68 (1.11)	4.00–8.00	10.32 (2.15)	4.00–13.00	71.00 (23.92)	16.00–104.00
	Female (n = 33)	6.58 (1.23)	4.00–8.00	9.27 (2.10)	5.00–12.00	63.12 (23.13)	20.00–96.00
30–39	Total (n = 31)	6.00 (1.44)	3.00–8.00	9.00 (2.29)	4.00–13.00	57.03 (24.67)	12.00–104.00
	Male (n = 15)	6.53 (1.19)	4.00–8.00	9.87 (1.85)	6.00–13.00	66.33 (22.75)	24.00–104.00
	Female (n = 16)	5.50 (1.51)	3.00–8.00	8.19 (2.43)	4.00–11.00	48.31 (23.81)	12.00–88.00
40–49	Total (n = 31)	5.97 (1.08)	4.00–8.00	8.68 (1.81)	5.00–12.00	53.52 (19.77)	20.00–96.00
	Male (n = 15)	6.13 (1.06)	4.00–8.00	9.00 (1.89)	5.00–12.00	56.93 (20.50)	20.00–96.00
	Female (n = 16)	5.81 (1.11)	4.00–8.00	8.38 (1.75)	5.00–12.00	50.31 (19.15)	20.00–96.00
50–59	Total (n = 34)	5.68 (1.17)	4.00–8.00	7.94 (2.00)	4.00–11.00	47.09 (20.54)	16.00–88.00
	Male (n = 15)	5.67 (1.11)	4.00–8.00	7.60 (2.03)	5.00–11.00	44.93 (19.96)	20.00–88.00
	Female (n = 19)	5.68 (1.25)	4.00–8.00	8.21 (1.99)	4.00–11.00	48.79 (21.38)	16.00–88.00
60–69	Total (n = 29)	5.00 (1.07)	3.00–7.00	6.83 (2.16)	3.00–12.00	36.17 (17.75)	9.00–84.00
	Male (n = 15)	5.47 (0.99)	3.00–7.00	7.80 (1.90)	4.00–12.00	44.20 (16.82)	12.00 −84.00
	Female (n = 14)	4.50 (0.94)	3.00–6.00	5.79 (1.97)	3.00–10.00	27.57 (14.84)	9.00–60.00

**TABLE 3:** Descriptive data for the letter-number sequencing task (i.e., verbal working memory): span length (longest correctly remembered sequence, range: 2–8), raw score (number of correctly remembered trials, range: 0–30), and product score (compound score of span length and raw score, range: 0–240).

	Verbal working memory						
		Span length		Raw score		Product score	
Decade	N	Mean (SD)	Range	Mean (SD)	Range	Mean (SD)	Range
All	Total (n = 184)	5.83 (0.98)	4.00–8.00	20.20 (2.47)	15.00–28.00	119.59 (33.23)	60.00–224.00
	Male (n = 85)	6.02 (1.02)	4.00–8.00	20.59 (2.71)	15.00–28.00	126.24 (36.27)	60.00–224.00
	Female (n = 99)	5.66 (0.92)	4.00–8.00	19.87 (2.19)	15.00–25.00	113.89 (29.38)	60.00–200.00
18–29 years	Total (n = 58)	5.90 (0.93)	4.00–8.00	20.60 (2.58)	16.00–28.00	123.34 (33.84)	64.00–224.00
	Male (n = 25)	6.20 (1.00)	5.00–8.00	21.12 (2.98)	16.00–28.00	133.24 (39.14)	80.00–224.00
	Female (n = 33)	5.67 (0.82)	4.00–7.00	20.21 (2.20)	16.00–25.00	115.85 (27.51)	64.00–175.00
30–39 years	Total (n = 31)	6.26 (1.03)	5.00–8.00	21.55 (2.17)	18.00–26.00	136.61 (34.26)	90.00–200.00
	Male (n = 15)	6.80 (0.94)	5.00–8.00	22.53 (1.92)	19.00–26.00	154.40 (31.55)	105.00–200.00
	Female (n = 16)	5.75 (0.86)	5.00–7.00	20.62 (2.03)	18.00–24.00	119.94 (28.37)	90.00–168.00
40–49 years	Total (n = 31)	5.84 (0.93)	4.00–8.00	19.81 (2.48)	15.00–25.00	117.39 (31.77)	68.00–200.00
	Male (n = 15)	5.87 (0.83)	4.00–7.00	19.87 (2.67)	15.00–24.00	118.20 (30.20)	68.00–168.00
	Female (n = 16)	5.81 (1.05)	5.00–8.00	19.75 (2.38)	16.00–25.00	116.62 (34.16)	80.00–200.00
50–59 years	Total (n = 34)	5.76 (0.99)	4.00–8.00	19.91 (2.01)	16.00–23.00	116.15 (29.80)	72.00–184.00
	Male (n = 15)	5.40 (0.99)	4.00–8.00	19.53 (2.03)	16.00–23.00	106.93 (29.97)	72.00–184.00
	Female (n = 19)	6.05 (0.91)	5.00–8.00	20.21 (1.99)	17.00–23.00	123.42 (28.33)	90.00–184.00
60–69 years	Total (n = 30)	5.30 (0.88)	4.00–7.00	18.77 (2.19)	15.00–23.00	100.93 (27.02)	60.00–154.00
	Male (n = 15)	5.73 (0.88)	4.00–7.00	19.53 (2.56)	15.00–23.00	113.73 (29.78)	60.00–154.00
	Female (n = 15)	4.78 (0.64)	4.00–6.00	18.00 (1.46)	15.00–20.00	88.13 (16.55)	60.00–114.00

Stepwise multiple regression analyses were performed to determine the most important predictors for visual (Table 4) and verbal (Table 5) span length, raw score, and product score. For all analyses, assumptions to appropriately conduct stepwise multiple regression were met, except for the presence of significant outliers. Specifically, each cognitive outcome variable showed significant outliers, ranging from one to maximal three outliers per variable. These outliers did not show large leverage values and/or influence and were therefore considered as reliable data points. Moreover, analysis with and without outliers showed similar results and therefore the outliers were not excluded from the analyses (Laerd Statistics, 2015).

**TABLE 4:** Stepwise multiple regression with age, sex, educational level, awakeness, and hearing sensitivity as independent variables and visual working memory (span length, raw score, and product score) as dependent variable.

	Visual working memory							
	B	SE B	β	t	p	F	R^2^	R^2^ adjusted
Model 1: span length	–	–	–	–	<0.001	17.294	0.225	0.212
**Significant predictors**								
Constant	6.114	0.689	–	8.869	<0.001	–	–	–
Age	−0.033	0.006	−0.393	−5.857	<0.001	–	–	–
Sex	−0.452	0.172	−0.174	−2.626	0.009	–	–	–
Educational level	0.095	0.039	0.164	2.427	0.016	–	–	–
**Excluded variables**								
Awakeness	–	–	−0.094	−1.377	0.170	–	–	–
Hearing sensitivity	–	–	−0.082	−0.918	0.360	–	–	–
Model 2: raw score	–	–	–	–	<0.001	21.002	0.260	0.248
**Significant predictors**								
Constant	9.324	1.197	–	7.787	<0.001	–	–	–
Age	−0.064	0.010	−0.423	−6.453	<0.001	–	–	–
Sex	−1.004	0.299	−0.217	−3.358	0.001	–	–	–
Educational level	0.159	0.068	0.155	2.344	0.020	–	–	–
**Excluded variables**								
Awakeness	–	–	0.051	−0.766	0.445	–	–	–
Hearing sensitivity	–	–	−0.114	−1.302	0.195	–	–	–
Model 3: product score	–	–	–	–	<0.001	20.175	0.253	0.240
**Significant predictors**								
Constant	60.341	12.501	–	4.827	<0.001	–	–	–
Age	−0.661	0.103	−0.422	−6.399	<0.001	–	–	–
Sex	−9.354	3.122	−0.195	−2.996	0.003	–	–	–
Educational level	1.678	0.707	0.157	2.373	0.019	–	–	–
**Excluded variables**								
Awakeness	–	–	−0.064	−0.957	0.340	–	–	–
Hearing sensitivity	–	–	−0.099	−1.128	0.261	–	–	–

*p < 0.05, significant; B, unstandardized regression coefficient; SE B, standard error of the coefficient; β, standardized coefficient; R^2^, coefficient of determination; –, not applicable.*

**TABLE 5:** Stepwise multiple regression with age, sex, educational level, awakeness, and hearing sensitivity as independent variables and verbal working memory (span length, raw score, and product score) as dependent variable.

	Verbal working memory							
	B	SE B	β	T	p	F	R^2^	R^2^ adjusted
Model 1: span length	–	–	–	–	<0.001	7.644	0.113	0.098
**Significant predictors**								
Constant	5.251	0.556	–	9.436	<0.001	–	–	–
Age	−0.011	0.005	−0.174	−2.426	0.016	–	–	–
Sex	−0.420	0.139	−0.214	−3.025	0.003	–	–	–
Educational level	0.081	0.031	0.187	2.597	0.010	–	–	–
**Excluded variables**						–	–	–
Awakeness	–	–	0.017	0.227	0.820	–	–	–
Hearing sensitivity	–	–	0.064	0.664	0.508	–	–	–
Model 2: raw score	–	–	–	–	<0.001	11.605	0.162	0.148
**Significant predictors**								
Constant	17.859	1.359	–	13.144	<0.001	–	–	–
Educational level	0.280	0.077	0.257	3.661	<0.001	–	–	–
Age	−0.037	0.011	−0.228	−3.274	0.001	–	–	–
Sex	−0.899	0.339	−0.182	−2.654	0.009	–	–	–
**Excluded variables**								
Awakeness	–	–	−0.014	−0.195	0.846	–	–	–
Hearing sensitivity	–	–	0.047	0.509	0.611	–	–	–
Model 3: product score	–	–	–	–	<0.001	10.405	0.148	0.134
**Significant predictors**								
Constant	91.903	18.458	–	4.979	<0.001			
Educational level	3.451	1.040	0.235	3.317	0.001	–	–	–
Sex	−14.538	4.602	−0.219	−3.159	0.002	–	–	–
Age	−0.434	0.152	−0.200	−2.847	0.005	–	–	–
**Excluded variables**								
Awakeness	–	–	−0.008	−0.114	0.909	–	–	–
Hearing sensitivity	–	–	0.054	0.573	0.568	–	–	–

*p < 0.05, significant, B, unstandardized regression coefficient; SE B, standard error of the coefficient; β, standardized coefficient; R^2^, coefficient of determination; –, not applicable.*

For visual span length [*F*(3,179) = 17.294, *p* < 0.001], raw score [*F*(3,180) = 11.605, *p* < 0.001], and product score [*F*(3,179) = 20.175, *p* < 0.001], the models were statistically significant and predicted, respectively, 21.2, 24.8, and 24.0% of the variance. For these three dependent variables, age, sex, and educational level were significant predictors, whereas awakeness and hearing sensitivity were not. Specifically, advancing age and lower educational levels were associated with lower scores on all outcome variable measuring visual working memory. Regarding sex, males outperformed females.

The models predicting verbal span length [*F*(3,180) = 7.644, *p* < 0.001], raw score [*F*(3,179) = 21.002, *p* < 0.001], and product score [*F*(3,180) = 10.405, *p* < 0.001] were statistically significant and predicted, respectively, 9.8, 14.8, and 13.4% of the variance. For these three dependent variables, age, sex, and educational level were significant predictors, whereas awakeness and hearing sensitivity were not. Specifically, advancing age and lower educational levels were associated with lower scores on all outcome variable measuring verbal working memory. Regarding sex, males performed better than females.

### Processing Speed

**FIGURE 1:**
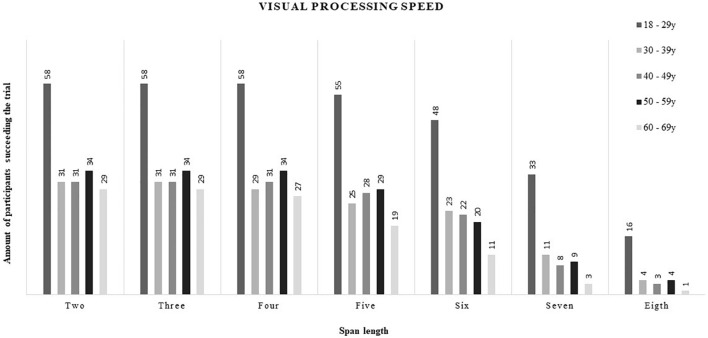
Visual processing speed.

**FIGURE 2:**
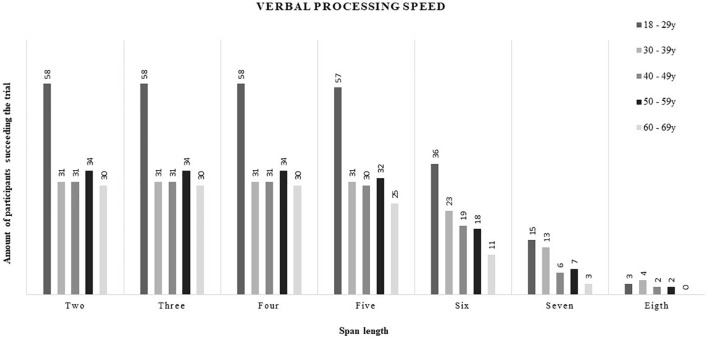
Verbal processing speed.

Descriptive data (mean, SD, and range) for visual and verbal processing speed at each span length are presented in Tables 6, 7, respectively. Percentile distributions of these outcome variables are presented in digital Supplementary Tables 3, 4. In general, an increase in visual and verbal processing speed as well as an increase in standard deviation among the results were observed with increasing span length. As can be seen in Figures 1, 2, the amount of participants succeeding trials with increasing span length diminished, especially within the older adults, for, respectively, visual and verbal processing speed.

**TABLE 6:** Descriptive data for visual processing speed (ms) of the backward corsi tapping task.

Visual processing speed															
		Span length 2		Span length 3		Span length 4		Span length 5		Span length 6		Span length 7		Span length 8	
Decade		Mean (SD)	Range	Mean (SD)	Range	Mean (SD)	Range	Mean (SD)	Range	Mean (SD)	Range	Mean (SD)	Range	Mean (SD)	Range
All	Total	656 (234)	121–1,917	1,245 (320)	551–2,771	2,005 (496)	958–3,956	2,695 (683)	1,706–6,942	3,249 (968)	1,781–7,773	5,146 (3,104)	2,345–22,283	4,949 (1,288)	2,840–7,694
	Male	652 (257)	121–1,917	1,248 (277)	647–1,958	2,011 (451)	1,124–3,956	2,655 (524)	1,802–4,309	3,273 (1,040)	1,912–7,773	5,825 (3,888)	3,076–22,283	4,981 (1,228)	3,780–7,694
	Female	661 (213)	318–1,583	1,242 (354)	551–2,771	1,999 (535)	958–3,951	2,735 (813)	1,706–6,942	3,244 (896)	1,781–5,478	4,507 (1,982)	2,345 (12,982)	4,916 (1,391)	2,840–6,742
18−29	Total	544 (167)	300–1,032	1,065 (253)	551–1,871	1,704 (322)	958–2,680	2,409 (542)	1,706–4,402	2,983 (858)	1,781–5,478	4,857 (3,780)	2,345–22,283	4,453 (1,217)	2,840–7,694
	Male	536 (184)	300–1,032	1,062 (270)	647–1,871	1,696 (322)	1,124–2,531	2,363 (421)	1,803–3,271	2,989 (877)	2,099–5,382	6,399 (5,370)	3,324–22,283	4,840 (1,314)	3,780–7,694
	Female	551 (156)	318–907	1,067 (244)	551–1,613	1,710 (327)	958–2,680	2,445 (624)	1,706–4,402	2,979 (859)	1,781–5,478	3,720 (1,169)	2,345–7,570	4,151 (1,116)	2,840–6,742
30−39	Total	585 (123)	387–922	1,149 (215)	783–1,758	1,831 (294)	1,415–2,929	2,399 (410)	1,802–3,877	2,888 (605)	1,912–4,317	6,073 (3,222)	3,076–12,982	5,129 (1,041)	4,105–6,274
	Male	528 (96)	387–721	1,158 (178)	783–1,457	1,822 (208)	1,537–2,289	2,286 (211)	1,802–2,643	2,935 (610)	1,912–4,317	5,568 (2,827)	3,076–11,492	4,747 (866)	4,105–5,732
	Female	639 (123)	425–922	1,141 (251)	829–1,758	1,840 (374)	1,415–2,929	2,543 (553)	1,892–3,877	2,828 (625)	2,062–4,178	6,958 (4,120)	3,664–12,982	6,274 (−)	6,274–6,274
40−49	Total	701 (241)	367–1,583	1,268 (248)	744 (1,702)	2,009 (310)	1,315–2,596	2,727 (466)	2,056 (4,174)	3,184 (749)	2,324 (5,252)	4,693 (868)	3,518–6,291	4,866 (895)	4,043–5,819
	Male	682 (205)	401–1,112	1,262 (206)	893–1,702	1,998 (259)	1,586–2,510	2,614 (288)	2,299–3,176	2,981 (436)	2,363–3,585	4,505 (537)	3,857–5,070	4,390 (491)	4,043–4,737
	Female	719 (276)	367–1,583	1,273 (289)	744–1,681	2,018 (360)	1,315–2,596	2,841 (584)	2,056–4,174	3,429 (977)	2,324–5,252	4,882 (1,173)	3,518–6,291	5,819 (−)	5,819–5,819
50−59	Total	738 (276)	383–1,917	1,343 (307)	819–2,316	2,223 (503)	1,355–3,956	2,940 (490)	1,902–3,966	3,867 (909)	2,859–6,473	5,564 (1,691)	3,178–9,296	6,453 (916)	5,321–7,509
	Male	814 (347)	482–1,917	1,388 (242)	1,039–1,958	2,395 (599)	1,737–3,956	3,034 (473)	2,181–3,966	4,032 (1,122)	3,173–6,473	5,900 (1,999)	4,401–9,296	6,415 (1,547)	5,321–7,509
	Female	678 (193)	383–1,157	1,307 (352)	819–2,316	2,088 (375)	1,355–2,751	2,863 (505)	1,902–3,564	3,758 (771)	2,859–5,460	5,145 (1,367)	3,178 (6,095)	6,490 (342)	6,248 (6,732)
60−69	Total	814 (252)	121–1,321	1,567 (333)	1,137–2,771	2,558 (560)	1,684–3,951	3,489 (1,040)	2,435–6,942	4,164 (1,479)	2,684–7,773	4,873 (1,002)	4,112–6,009	6,395 (−)	6,395–6,395
	Male	776 (284)	121–1,321	1,492 (215)	1,137–1,865	2,356 (272)	2,064–2,926	3,255 (432)	2,741–4,309	4,283 (1,624)	2,684–7,773	4,499 (−)	4,499–4,499	–	–
	Female	854 (216)	459–1,266	1,648 (419)	1,146–2,771	2,809 (723)	1,684–3,951	3,998 (1,730)	2,435–6,942	3,847 (1,220)	2,714–5,139	5,061 (1,341)	4,112–6,009	6,395 (−)	6,395–6,395

**TABLE 7:** Descriptive data for verbal processing speed (ms) of the letter-number sequencing task.

Verbal processing speed															
		Span length 2		Span length 3		Span length 4		Span length 5		Span length 6		Span length 7		Span length 8	
Decade		Mean (SD)	Range	Mean (SD)	Range	Mean (SD)	Range	Mean (SD)	Range	Mean (SD)	Range	Mean (SD)	Range	Mean (SD)	Range
All	Total	783 (225)	320–1,960	1,629 (539)	460–4,360	2,770 (1,055)	1,130–6,590	4,810 (2,757)	1,160–25,280	7,041 (2,833)	2,060–15,560	9,193 (3,769)	3,990–19,500	10,007 (3,954)	3,160–16,650
	Male	754 (216)	330–1,520	1,529 (484)	460–2,760	2,670 (1,155)	1,130–6,590	4,450 (2,185)	1,160–15,060	6,761 (3,075)	2,060–15,560	8,356 (3,302)	3,990–17,560	10,444 (4,367)	3,160–16,650
	Female	807 (231)	320–1,960	1,715 (571)	710–4,360	2,857 (959)	1,450–5,920	5,120 (3,147)	1,560–25,280	7,372 (2,507)	3,410–13,490	10,294 (4,141)	4,130–19,500	8,843 (2,938)	5,990–11,860
18−29 years	Total	755 (235)	320–1,330	1,426 (450)	460–2,710	2,591 (871)	1,130–5,280	4,314 (2,334)	1,160–15,060	6,819 (2,710)	2,060–13,490	9,701 (3,470)	3,990–16,400	6,493 (3,839)	3,160–10,690
	Male	743 (283)	330–1,330	1,413 (479)	460–2,130	2,542 (901)	1,130–4,800	4,355 (2,760)	1,160–15,060	6,668 (2,583)	2,060–11,250	9,072 (3,077)	3,990–13,560	6,493 (3,839)	3,160–10,690
	Female	763 (194)	320–1,140	1,436 (435)	710–2,710	2,628 (859)	1,450–5,280	4,281 (1,985)	1,560–11,690	6,970 (2,897)	3,410–13,490	10,643 (4,096)	4,650–16,400	–	–
30−39 years	Total	765 (177)	510–1,200	1,449 (385)	650–2,180	2,288 (569)	1,460–4,140	4,069 (1,497)	2,130–8,650	7,912 (3,452)	3,390–15,560	9,703 (4,137)	5,300–17,560	12,388 (2,915)	10,250–16,650
	Male	706 (124)	510–880	1,327 (387)	650–2,030	2,047 (474)	1,460–3,290	3,533 (1,131)	2,130–5,830	7,626 (4,109)	3,390–15,560	8,947 (3,998)	5,300–17,560	12,388 (2,915)	10,250−16,650
	Female	821 (203)	550–1,200	1,564 (358)	1,050–2,180	2,514 (570)	1,790–4,140	4,571 (1,653)	2,290–8,650	8,357 (2,227)	5,800–12,810	11,405 (4,500)	5,800–15,730	–	–
40−49 years	Total	802 (217)	420–1,220	1,775 (473)	730–2,760	2,730 (923)	1,320–5,220	5,131 (2,251)	1,820–9,990	7,086 (3,071)	3,280–15,530	6,438 (2,027)	4,010–8,630	7,335 (1,902)	5,990–8,680
	Male	743 (191)	420–1,160	1,676 (544)	730–2,760	2,506 (987)	1,320–5,220	5,011 (2,177)	1,820–8,200	7,316 (3,577)	3,280–15,530	6,473 (2,280)	4,010–8,510	–	–
	Female	857 (230)	550–1,220	1,868 (391)	1,050–2,490	2,940 (836)	1,560–4,560	5,237 (2,381)	2,120–9,990	6,769 (2,403)	3,720–10,830	6,403 (2,250)	4,130–8,630	7,335 (1,902)	5,990–8,680
50–59 years	Total	817 (210)	380–1,520	1,693 (461)	850–2,660	2,927 (1,148)	1,380–6,590	5,366 (4,096)	2,490–25,280	7,120 (2,498)	3,650–12,580	10,614 (4,290)	7,080–19,500	13,190 (1,881)	11,860–14,520
	Male	828 (224)	580–1,520	1,635 (481)	940–2,660	3,075 (1,259)	1,380–6,590	4,629 (1,813)	2,920–9,520	5,633 (2,495)	3,650–10,150	7,410 (−)	7,410–7,410	14,520 (−)	14,520–14,520
	Female	808 (203)	380–1,250	173 (452)	850–2,600	2,811 (1,072)	1,590–5,710	5,870 (5,104)	2,490–25,280	7,863 (2,238)	5,130–12,580	11,148 (4,438)	7,080–19,500	11,860 (−)	11,860–11,860
60–69 years	Total	797 (279)	430–1,960	1,985 (729)	830–4,360	3,479 (1,399)	1,350–6,400	5,764 (3,002)	2,610–15,580	5,737 (1,331)	4,260–8,060	6,630 (3,118)	4,250–10,160	–	–
	Male	759 (178)	450–1,130	1,670 (457)	920–2,600	3,265 (1,672)	1,350–6,400	4,875 (2,147)	2,610–9,560	5,676 (1,360)	4,260–8,060	6,630 (3,118)	4,250–10,160	–	–
	Female	835 (355)	430–1,960	2,299 (825)	830–4,360	3,693 (1,077)	2,450–5,920	6,896 (3,622)	3,240–15,580	6,015 (1,648)	4,850–7,180	–	–	–	–

Stepwise multiple regression analyses were performed to determine the most important predictors for visual (Table 8) and verbal (Table 9) processing speed at each span length. For all analyses, assumptions to appropriately conduct stepwise multiple regression were met, except for the presence of significant outliers. Specifically, each cognitive outcome variable showed significant outliers, ranging from one to maximal four outliers per variable. As with working memory, outliers were considered reliable (i.e., no large leverage values and/or influence) and were not excluded from the analyses (Laerd Statistics, 2015).

**TABLE 8:** Stepwise multiple regression with age, sex, educational level, awakeness, and hearing sensitivity as independent variables and visual processing speed at each span length as dependent variable.

	Visual processing speed							
	B	SE B	β	t	p	F	R^2^	R^2^ adjusted
Model 1: span length 2	–	–	–	–	<0.001	24.784	0.216	0.207
**Significant predictors**								
Constant	416.860	46.236	–	9.016	<0.001	–	–	–
Age	4.548	1.361	0.298	3.341	0.001	–	–	–
Hearing sensitivity	8.642	3.678	0.209	2.349	0.020	–	–	–
**Excluded variables**								
Sex	–	–	0.050	0.753	0.453	–	–	–
Educational level	–	–	−0.082	−1.213	0.227	–	–	–
Awakeness	–	–	−0.042	−0.615	0.539	–	–	–
Model 2: span length 3	–	–	–	–	<0.001	38.561	0.300	0.292
**Significant predictors**								
Constant	826.645	59.722	–	13.841	<0.001	–	–	–
Age	8.806	1.758	0.421	5.008	<0.001	–	–	–
Hearing sensitivity	9.425	4.751	0.167	1.984	0.049	–	–	–
**Excluded variables**								
Sex	–	–	0.024	0.385	0.701	–	–	–
Educational level	–	–	−0.023	−0.357	0.721	–	–	–
Awakeness	–	–	−0.040	−0.613	0.540	–	–	–
Model 3: span length 4	–	–	–	–	<0.001	37.607	0.392	0.382
**Significant predictors**								
Constant	1,737.677	236.601	–	7.344	<0.001	–	–	–
Age	14.549	2.559	0.448	5.686	<0.001	–	–	–
Hearing sensitivity	16.971	7.014	0.190	2.420	0.017	–	–	–
Educational level	−27.650	13.433	−0.124	−2.057	0.041	–	–	–
**Excluded variables**								
Sex	–	–	0.051	0.852	0.395	–	–	–
Awakeness	–	–	−0.035	−0.568	0.571	–	–	–
Model 4: span length 5	–	–	–	–	<0.001	30.140	0.283	0.273
**Significant predictors**								
Constant	1,858.353	137.747	–	13.491	<0.001	–	–	–
Age	17.734	4.072	0.385	4.356	<0.001	–	–	–
Hearing sensitivity	24.114	10.828	0.197	2.227	0.027	–	–	–
**Excluded variables**						–	–	–
Sex	–	–	0.133	1.938	0.054	–	–	–
Educational level	–	–	−0.070	−1.006	0.316	–	–	–
Awakeness	–	–	0.051	0.716	0.475	–	–	–
Model 5: span length 6	–	–	–	–	<0.001	14.528	0.194	0.180
**Significant predictors**								
Constant	2,319.690	231.971	–	10.000	<0.001	–	–	–
Age	19.466	7.025	0.282	2.771	0.006	–	–	–
Hearing sensitivity	38.252	18.609	0.209	2.056	0.042	–	–	–
**Excluded variables**								
Sex	–	–	0.026	0.317	0.752	–	–	–
Educational level	–	–	−0.075	−0.908	0.366	–	–	–
Awakeness	–	–	−0.007	−0.084	0.933	–	–	–
Model 6: span length 7	No variables were entered into the equation							
Model 7: span length 8	–	–	–	–	0.010	7.609	0.226	0.197
**Significant predictors**								
Constant	3,326.018	627.379	–	5.301	<0.001	–	–	–
Age	49.490	17.941	0.476	2.758	0.010	–	–	–
**Excluded variables**								
Sex	–	–	−0.034	−0.193	0.848	–	–	–
Educational level	–	–	−0.256	−1.478	0.152	–	–	–
Awakeness	–	–	−0.271	−1.600	0.122	–	–	–
Hearing sensitivity	–	–	−0.302	−1.488	0.149	–	–	–

*p < 0.05, significant; B, unstandardized regression coefficient; SE B, standard error of the coefficient; β, standardized coefficient; R^2^, coefficient of determination; –, not applicable.*

**TABLE 9:** Stepwise multiple regression with age, sex, educational level, awakeness, and hearing sensitivity as independent variables and verbal processing speed at each span length as dependent variable.

	Verbal processing speed							
	B	SE B	β	t	p	F	R^2^	R^2^ adjusted
Model 1: span length 2	–	–	–	–	0.014	6.194	0.033	0.028
**Significant predictors**								
Constant	737.522	24.491	–	30.144	<0.001	–	–	–
Hearing sensitivity	7.217	2.900	0.181	2.489	0.014	–	–	–
**Excluded variables**						–	–	–
Age	–	–	−0.049	−0.496	0.620	–	–	–
Sex	–	–	0.133	1.827	0.069	–	–	–
Educational level	–	–	−0.064	−0.854	0.394	–	–	–
Hearing sensitivity	–	–	0.106	1.445	0.150	–	–	–
Model 2: span length 3	–	–	–	–	<0.001	12.649	0.220	0.203
**Significant predictors**								
Constant	1,692.162	287.137	–	5.893	<0.001	–	–	–
Age	7.605	3.152	0.216	2.413	0.017	–	–	–
Sex	231.168	71.762	0.214	3.221	0.002	–	–	–
Educational level	−39.558	16.255	−0.166	−2.434	0.016	–	–	–
Hearing sensitivity	17.840	8.560	0.188	2.084	0.039	–	–	–
**Excluded variables**								
Awakeness	–	–	0.053	0.780	0.436	–	–	–
Model 3: span length 4	–	–	–	–	<0.001	12.125	0.118	0.108
Significant predictors								
Constant	3,298.907	594.035	–	5.553	<0.001	–	–	–
Age	18.008	4.908	0.262	3.669	<0.001	–	–	–
Educational level	−81.995	33.330	−0.175	−2.460	0.015	–	–	–
**Excluded variables**								
Sex	–	–	0.119	1.697	0.091	–	–	–
Awakeness	–	–	0.017	0.236	0.814	–	–	–
Hearing sensitivity	–	–	0.091	0.959	0.339	–	–	–
Model 4: span length 5	–	–	–	–	6.960	0.009	0.039	0.033
** Significant predictors**								
Constant	3,364.092	585.185	–	5.749	<0.001	–	–	–
Age	36.013	13.651	0.197	2.638	0.009	–	–	–
Excluded variables								
Sex	–	–	0.130	1.746	0.083	–	–	–
Educational level	–	–	−0.089	−1.165	0.246	–	–	–
Awakeness	–	–	0.032	0.412	0.681	–	–	–
Hearing sensitivity	–	–	−0.048	−0.456	0.649	–	–	–
Model 5: span length 6	No variables were entered into the equation							
Model 6: span length 7	No variables were entered into the equation							
Model 7: span length 8	No variables were entered into the equation							

*p < 0.05, significant; B, unstandardized regression coefficient; SE B, standard error of the coefficient; β, standardized coefficient; R^2^, coefficient of determination; –, not applicable.*

The models predicting visual processing speed were statistically significant at span lengths two [*F*(2,180) = 24.784, *p* < 0.001], three [*F*(2,180) = 38.561, *p* < 0.001], four [*F*(3,175) = 37.607, *p* < 0.001], five [*F*(2,153) = 30.140, *p* < 0.001], six [*F*(2,121) = 14.528, *p* < 0.001], and eight [*F*(1,26) = 7.609, *p* = 0.010] and predicted, respectively, 20.7, 29.2, 38.2, 27.3, 18.0, and 19.7% of the variance. For visual processing speed at span length seven, all variables were excluded from the model. None of the significant models included awakeness and sex as significant predictors, whereas age was revealed as significant predictor within each significant model. Specifically, slower processing speed was associated with increasing age. In addition to age, hearing sensitivity was revealed as significant predictor for visual processing speed at span lengths two, three, five and six; i.e., the higher the average hearing loss on all measured frequencies, the slower visual processing speed will be. In addition to age and hearing sensitivity, educational level was revealed as significant predictor for visual processing speed, howbeit only at span length four. Lower educational levels were associated with slower visual processing speed.

For verbal processing speed at span length two [*F*(1,182) = 6.194, *p* = 0.014], three [*F*(4,179) = 12.649, *p* < 0.001], four [*F*(2,181) = 12.125, *p* < 0.001], and five [*F*(1,173) = 6.960, *p* = 0.009] the model was statistically significant and predicted, respectively, 2.8, 20.3, 10.8, and 3.3% of the variance. From span lengths six up to eight, none of the variables seemed to have a significant association with verbal processing speed, hence all variables were excluded from the model. In none of the significant models awakeness was revealed as significant predictor. Age was included in all significant models except for span length two. At span length two, hearing sensitivity was revealed as the only significant predictor for verbal processing speed. In addition to age, educational level contributed in predicting verbal processing speed at span length four. At span length three, age, sex, educational level, and hearing sensitivity were all revealed as important predictors for verbal processing speed. Overall, advancing age, lower educational levels, and worse hearing sensitivity were associated with slower verbal processing speed. Regarding sex, males performed better than females.

## Discussion

To strengthen future methodological choices regarding the measurement of cognition within the field of audiology, the current study aimed to examine the effect of, among other things, hearing sensitivity on visual and verbal tests to measure working memory and processing speed. A clear distinction between tasks in which the test items were presented visually and auditorily was made in order to rule out overlap in cognitive pathways as much as possible. However, participants’ internal strategy to solve the tasks might differ from the modality in which the test items were presented. Specifically, when conducting the backward corsi tapping task, it might be possible that a verbal code is used when encoding the places where the circles appeared in the raster (Vandierendonck et al., 2004). Likewise, when performing the letter-number sequencing task participants might utilize visualization of the verbal information (Haut et al., 2000). The used internal strategies to solve a particular task might differ across participants. Evaluating participants’ internal strategy was beyond the scope of the current research, though can be subject of future research that includes, among other things, functional neuroimaging techniques.

The current results indicated a significant association between hearing sensitivity and visual processing speed, whereas hearing sensitivity did not show any association with verbal working memory, verbal processing speed, and visual working memory. However, based on previous literature within hearing-impaired adults, the opposite was expected. It was assumed that, in addition to lower cognitive abilities due to comorbidity with hearing loss, verbal cognitive testing in hearing-impaired adults may negatively impact cognitive outcomes (Shen et al., 2016; Füllgrabe, 2020b). Specifically, the perceptual processing of auditory stimuli within hearing-impaired individuals might allocate more explicit and slower cognitive processing which may lead to fewer available cognitive resources that can be used toward the execution of the cognitive task itself. However, the authors are aware that the current study population consisted of relatively “young” older-adults with age-appropriate hearing. Hence, older-adults were allowed to show mild levels of high-frequency hearing loss. Hearing-impaired older-adults seeking audiological help might suffer from more hearing loss and will mostly have an age above 69 years. To verify if the lack in association between hearing sensitivity and verbal cognitive testing remains within adults with more hearing loss, further research should conduct a similar study design including hearing-impaired older-adults across a broader age span showing different levels of hearing loss. The underlying phenomenon why solely visual processing speed was associated with hearing sensitivity was unclear. Nevertheless, the current results significantly highlight the importance to further evaluate the effect of visual and verbal cognitive tests within audiological research and to evaluate their relation with speech understanding and listening effort. Specifically, based on cognitive construct validity it is hypothesized that verbal cognitive testing will better predict speech understanding, especially in noisy listening conditions, compared to visual testing (Vaughan et al., 2008). In this respect, the fact that verbal cognitive testing was not associated with hearing sensitivity can be seen as an additional incentive to implement verbal cognitive tests within audiological practice.

One possible application of measuring working memory and processing speed within audiological research is to conduct hearing aid fitting based on the auditory-cognitive performance of the hearing aid user. More specifically, hearing aid users with poorer cognitive functioning seemed to derive more hearing aid benefit in terms of speech understanding from hearing aid settings facilitating the matching process between the incoming auditory signal and representations stored in long-term memory (Kestens et al., 2021a). However, the boundary between good and poor cognitive functioning is not yet clearly defined in literature (Kestens et al., 2021a). Clinically, a score lower than the 5th percentile is commonly used as cutoff point for an impaired performance within neuropsychology (Lezak et al., 2004). However, if this cutoff point is also relevant within the field of audiology has to be further examined. Moreover, to be relevant for audiological research, a discrimination between good and poor cognitive performances should be able to make. Hence, the outcomes may not show any floor or ceiling effects but have to demonstrate sufficient variance.

### Working Memory

For both visual and verbal working memory, a ceiling effect was obtained for span length for almost all age decades, except for the oldest age group which is probably the most pertinent when it comes to a representative audiological patient populations. Product score and raw score demonstrated more variance among outcome scores, and might therefore, be considered as more valid outcome parameters. Moreover, the raw score takes into account all performances of trials of a similar span length, and thus is considered more valid than solely span length (Kessels et al., 2000). To determine which outcome variable will best suit implementation in audiological research, further research should focus on investigating their associations with speech understanding (in noise).

#### Visual Working Memory

In agreement with previous studies, span length, raw score, and product score significantly decreased with increasing age (e.g., Orsini et al., 1987; Kessels et al., 2008; Tamayo et al., 2012; Brunetti et al., 2014). In addition, males performed significantly better than females, likely due to sex differences in visuospatial processing (Weiss et al., 2003), which resulted in a general advantage of males over females in visuospatial tasks. This observed sex difference was in agreement with previous studies (Lewin et al., 2001; Tamayo et al., 2012; Brunetti et al., 2014), but contradicted Kessels et al. (2008) where no statistically significant sex difference was observed.

The current age-related data were comparable to those reported for adults aged 50 years or older by Kessels et al. (2008). However, the results reported by Brunetti et al. (2014) were lower compared to the current data. In the study of Brunetti et al. (2014), span length and raw score were computed for younger (mean age of 21.6 years) and older (mean age 57.6 years) adults. However, the specific age ranges of these groups were not mentioned. Moreover, a mean educational level of 14.8 years was reported in their study, which is a little lower compared to the mean educational level in the current study. Hence, the differences in age ranges and educational level explain the lower values compared to those reported in the current study.

#### Verbal Working Memory

Increasing age was related to a decrease in span length and raw score, which coincides with findings by Tamayo et al. (2012). As a result, a decrease in product score was also related to increasing age. Furthermore, a significant sex effect was observed, indicating that males performed better than females for span length, raw score, and product score. Contrastingly, previous research indicated a sex difference favoring females in verbal memory tasks (Lewin et al., 2001; Barel and Tzischinsky, 2018). Moreover, no sex difference for span length and raw score on the letter-number sequencing task was observed in previous studies (e.g., Irwing, 2012; Tamayo et al., 2012).

A reliable comparison with existing data is difficult to make. More specifically, the currently used Dutch version of the letter-number sequencing task has only normative data for span length, which were set up using other age groups and without sex distinction (WAIS-IV-NL). Moreover, the Spanish version of the letter-number sequencing task (Tamayo et al., 2012) computed span length and raw score, though a fewer amount of stimuli were used. Last, normative data for product score of the letter-number sequencing task are lacking in previous literature.

### Processing Speed

For both visual and verbal processing speed, descriptive data were computed per span length based on the best item out of the correctly solved items of that span length. These processing speed measures reflect speed under a working memory demand, which may be different than processing speed under other types of conditions (e.g., visual search or simple decision-making). Hence, a possible trade-off between the working memory and processing speed outcomes should be considered. Also, participant’s fastest processing speed score might not be the most suitable parameter to reveal impact in everyday activities. In this respect, the associations between processing speed outcomes and speech understanding (in noise) should be further investigated.

This study was the first to compute descriptive data for visual and verbal processing speed based on the backward corsi tapping task and letter-number sequencing task, respectively. Consequently, no comparison with previous literate could be made. Implementing processing speed of all span lengths into audiological research seems too extensive and might be unnecessary for several reasons. First, a decrease of participants succeeding trials with increasing span length was observed. More specifically, from approximately span length five, the amount of participants succeeding the trial started to diminish with most failures in older adults. This finding is in agreement with the abovementioned result of a decreased visual and verbal working memory with increasing age. Second, an increase in variance among the results was observed with increasing span length. Due to the diminished feasibility and greater variance among outcome scores of span length six or higher, the obtained results of visual and verbal processing speed at those higher span lengths might be less meaningful for audiological research. Third, based on practical experience, span lengths two and three seemed to be quite easy for both young- and older-adults. Hence, processing speed at span lengths four and five represented the best balance between too easy and too difficult, and thus, might suit best for implementation in audiological research. However, to make this suggestion hard, further research should focus on investigating the associations between processing speed measured at those span lengths and speech understanding (in noise). Only span lengths four and five are discussed below.

#### Visual Processing Speed

Increasing age negatively affected visual processing speed at, among others, span length four and five. Further, a significant sex effect, favoring males, was found at span length five. The latter might be related to sex differences in visuospatial processing (Weiss et al., 2003). Moreover, previous results did find a significant sex effect on a time-dependent outcome (i.e., reaction time) of the corsi backward tapping task (Brunetti et al., 2014). However, why this sex differences did not occur at span length four was unclear.

#### Verbal Processing Speed

Slower verbal processing speed was related to increasing age at span lengths four and five. No sex difference was found for verbal processing speed at span length four and five. Contrastingly, previous results indicated a faster and more accurate identification of alphabetical sequences in females compared to males (Majeres, 1997).

### Strengths and Limitations

This study is, to the best of our knowledge, the first to explore the use of visual and verbal working memory and processing speed data within audiological research. Moreover, current neuropsychological data regarding the backward corsi tapping task and letter-number sequencing task were expanded. The obtained percentile scores are preliminary and cannot be used as representative normative data for the entire population due to, among other things, lack of external validity.

Individuals with age-appropriate hearing from a broad age range (i.e., 18–69 years of age) were included. The authors are aware that mainly adults over the age of 69 with worse hearing sensitivity will be seeking audiological help and will benefit from auditory-cognitive testing. In line with the current results, it is expected that the performances on all outcomes parameters will further decrease with increasing age. However, the exact amount of decrement, in combination with hearing loss, is unknown and should be further explored.

Educational level was, among other things, a significant predictor for several outcome parameters, though only age and sex were taken into account when computing the descriptive data. Participants had a relative high educational level, and thus, computing descriptive data per educational level was considered not representative. Nevertheless, when using the currently obtained data, the possible influence of educational level should be kept in mind.

Within the current study, standardized approaches were used for both the backward corsi tapping task (Kessels et al., 2008) and the letter-number sequencing task (WAIS-III-NL), though these approaches differed in the amount of trials per span length. A digital version of the existing corsi tapping task (WAIS-III-NL) was used, hence identical stimulus presentation across participants and automatic data collection were guaranteed (Brunetti et al., 2014). The backward corsi tapping task was conducted using a wireless mouse and thus, performance on this task could have been influenced by participants’ motor and computer skills. Although a practice trial was implemented, one older adult had to be excluded for the backward corsi tapping task due to poor computer skills. Hence, working with a touch screen would be more beneficial. Furthermore, the distance between the screen and participant was not considered, but might be relevant for task performance. In further research this distance should be controlled. Trials of the letter-number sequencing task were presented through live voice, leading to a lower control of stimulus presentation compared to the backward corsi tapping task. Also, recall of presented sequences was required which complicates automatic computation of processing speed. Consequently, the usage of a stopwatch might have resulted in more human errors and greater variability across results compared to the automatic analysis of the backward corsi tapping task. Nevertheless, usage of a stopwatch for measuring time-related outcomes is commonly used within neuropsychological clinic, e.g., Trail-Making Test.

## Conclusion, Further Research, and Clinical Implications

The current results indicated a significant association between hearing sensitivity and visual processing speed, whereas hearing sensitivity was not associated with verbal working memory, verbal processing speed, and visual working memory. Hence, the current results highlight the importance to further evaluate cognitive construct validity within audiological research.

All outcome measures for visual and verbal working memory and processing speed showed a decrease in performance with increasing age. For visual and verbal working memory, males seemed to perform better compared to females, whereas no clear sex effect was observed for visual and verbal processing speed. The outcome parameters raw score and product score seem to be implementable measures of visual and verbal working memory. Concerning visual and verbal processing speed, only processing speed obtained at span length four and five might suit audiological implementation.

Further research can focus on expanding the current data by including older participants and participants showing more variety in hearing sensitivity and educational level. As such, normative data can be calculated. Moreover, the possible relationships between speech understanding and listening effort, and visual or verbal working memory and processing speed need to be explored. The latter should be conducted in both an auditory and auditory-visual context. Hence, it will be elucidated which cognitive functions should be useful to measure in audiological practice, which ultimately will emphasize audiological patient-centered care.

## Data Availability Statement

The raw data supporting the conclusions of this article will be made available by the authors, without undue reservation.

## Ethics Statement

The studies involving human participants were reviewed and approved by Ethics Committee Ghent University Hospital Corneel Heymanslaan 10 9000 Ghent. The patients/participants provided their written informed consent to participate in this study.

## Author Contributions

KK performed the data collection, data analysis, and the writing of the manuscript. SD, MM, and HK coordinated and supervised the data collection, and critically reviewed the manuscript. All authors substantially contributed to the manuscript.

## Conflict of Interest

The authors declare that the research was conducted in the absence of any commercial or financial relationships that could be construed as a potential conflict of interest.

## Publisher’s Note

All claims expressed in this article are solely those of the authors and do not necessarily represent those of their affiliated organizations, or those of the publisher, the editors and the reviewers. Any product that may be evaluated in this article, or claim that may be made by its manufacturer, is not guaranteed or endorsed by the publisher.
