# Supplementary material for: Visual and Verbal Working Memory and Processing Speed Across the Adult Lifespan: The Effect of Age, Sex, Educational Level, Awakeness, and Hearing Sensitivity
Source: Front Psychol. 2021 Oct 14;12:668828. doi: 10.3389/fpsyg.2021.668828 (PMC8551836; doi:10.3389/fpsyg.2021.668828)
Supplement: Supplementary file 1 [file Table_1.docx]

Supplemental Digital Table 1: Percentile distribution for the backward corsi tapping task (i.e. visual working memory) per decade and for males and females separately; span length: longest correctly remembered sequence, range: 2 – 8; raw score i.e. number of correctly remembered trials, range: 0 – 14; product score i.e. compound score of span length and raw score, range: 0 - 112.

| **VISUAL WORKING MEMORY** | | | | | | | | | | |
| --- | --- | --- | --- | --- | --- | --- | --- | --- | --- | --- |
| Decade | 18-29 | | 30-39 | | 40-49 | | 50-59 | | 60-69 | |
| Sex | Male | Female | Male | Female | Male | Female | Male | Female | Male | Female |
| N (%) | 25 (100.00%) | 33 (100.00%) | 15 (100.00%) | 16 (100.00%) | 15 (100.00%) | 16 (100.00%) | 15 (100.00%) | 19 (100.00%) | 15 (100.00%) | 14 (93.33%) |
| **SPAN LENGTH** | | | | | | | | | | |
| Pc 5 | 4.3 | 4.0 | 4.0 | 3.0 | 4.0 | 4.0 | 4.0 | 4.0 | 3.0 | 3.0 |
| Pc 10 | 5.0 | 5.0 | 4.6 | 3.0 | 4.6 | 4.0 | 4.0 | 4.0 | 3.6 | 3.0 |
| Pc 20 | 6.0 | 5.0 | 6.0 | 4.0 | 5.2 | 5.0 | 5.0 | 4.0 | 5.0 | 4.0 |
| Pc 30 | 6.0 | 6.0 | 6.0 | 4.1 | 6.0 | 5.0 | 5.0 | 5.0 | 5.0 | 4.0 |
| Pc 40 | 6.0 | 6.0 | 6.0 | 5.8 | 6.0 | 5.8 | 5.0 | 5.0 | 5.4 | 4.0 |
| Pc 50 | 7.0 | 7.0 | 6.0 | 6.0 | 6.0 | 6.0 | 6.0 | 6.0 | 6.0 | 4.5 |
| Pc 60 | 7.0 | 7.0 | 7.0 | 6.0 | 6.0 | 6.0 | 6.0 | 6.0 | 6.0 | 5.0 |
| Pc 70 | 7.2 | 7.0 | 7.2 | 6.0 | 6.2 | 6.0 | 6.0 | 6.0 | 6.0 | 5.0 |
| Pc 80 | 8.0 | 8.0 | 8.0 | 7.0 | 7.0 | 7.0 | 6.8 | 7.0 | 6.0 | 5.0 |
| Pc 90 | 8.0 | 8.0 | 8.0 | 7.3 | 8.0 | 7.3 | 7.4 | 8.0 | 6.4 | 6.0 |
| **RAW SCORE** | | | | | | | | | | |
| Pc 5 | 5.2 | 5.7 | 6.0 | 4.0 | 5.0 | 5.0 | 5.0 | 4.0 | 4.0 | 3.0 |
| Pc 10 | 8.0 | 6.0 | 7.2 | 4.0 | 5.6 | 5.7 | 5.0 | 5.0 | 4.6 | 3.5 |
| Pc 20 | 8.2 | 7.0 | 8.2 | 5.4 | 8.0 | 6.8 | 5.2 | 7.0 | 6.2 | 4.0 |
| Pc 30 | 9.0 | 8.0 | 9.0 | 6.2 | 8.0 | 8.0 | 6.0 | 7.0 | 7.8 | 4.5 |
| Pc 40 | 10.0 | 9.0 | 9.0 | 8.0 | 9.0 | 8.0 | 7.0 | 8.0 | 8.0 | 5.0 |
| Pc 50 | 10.0 | 10.0 | 10.0 | 9.0 | 9.0 | 8.0 | 7.0 | 8.0 | 8.0 | 5.5 |
| Pc 60 | 11.0 | 10.0 | 10.6 | 10.0 | 9.0 | 9.0 | 8.0 | 9.0 | 8.0 | 6.0 |
| Pc 70 | 12.0 | 10.8 | 11.0 | 10.0 | 10.2 | 9.0 | 9.2 | 9.0 | 8.0 | 6.5 |
| Pc 80 | 12.0 | 11.2 | 11.8 | 10.0 | 11.0 | 10.0 | 10.0 | 11.0 | 8.8 | 7.0 |
| Pc 90 | 13.0 | 12.0 | 12.4 | 11.0 | 11.4 | 10.6 | 10.4 | 11.0 | 10.8 | 9.5 |
| **PRODUCT SCORE** | | | | | | | | | | |
| Pc 5 | 23.2 | 22.8 | 24.0 | 24.0 | 20.0 | 20.0 | 20.0 | 16.0 | 12.0 | 9.0 |
| Pc 10 | 40.0 | 30.0 | 33.6 | 33.6 | 26.0 | 22.8 | 20.0 | 20.0 | 16.8 | 10.5 |
| Pc 20 | 54.0 | 40.0 | 49.2 | 49.2 | 41.6 | 34.0 | 26.0 | 32.0 | 31.0 | 16.0 |
| Pc 30 | 55.6 | 48.0 | 54.0 | 54.0 | 48.0 | 40.0 | 30.0 | 35.0 | 39.0 | 18.0 |
| Pc 40 | 60.0 | 57.6 | 56.4 | 56.4 | 54.0 | 46.4 | 35.0 | 40.0 | 43.2 | 20.0 |
| Pc 50 | 70.0 | 70.0 | 60.0 | 60.0 | 54.0 | 48.0 | 42.0 | 48.0 | 48.0 | 24.5 |
| Pc 60 | 77.0 | 70.8 | 71.4 | 71.4 | 54.0 | 54.0 | 48.0 | 54.0 | 48.0 | 30.0 |
| Pc 70 | 86.4 | 79.4 | 79.2 | 79.2 | 63.4 | 59.4 | 60.0 | 54.0 | 48.0 | 32.5 |
| Pc 80 | 96.0 | 84.8 | 94.4 | 94.4 | 77.0 | 67.2 | 62.4 | 77.0 | 52.8 | 35.0 |
| Pc 90 | 104.0 | 96.0 | 99.2 | 99.2 | 91.2 | 77.8 | 77.2 | 88.0 | 69.6 | 57.0 |
| Note. Pc = percentile | | | | | | | | | | |

Supplemental Digital Table 2: Percentile distribution for the letter-number sequencing task (verbal working memory) per decade and for males and females separately; span length: longest correctly remembered sequence, range: 2 – 8; raw score i.e. number of correctly remembered trials, range 0 – 30; ; product score i.e. compound score of span length and raw score, range: 0 – 240.

| **VERBAL WORKING MEMORY** | | | | | | | | | | | | |
| --- | --- | --- | --- | --- | --- | --- | --- | --- | --- | --- | --- | --- |
| Decade | 18-29 | | 30-39 | | 40-49 | | 50-59 | | | 60-69 | | |
| Sex | Male | Female | Male | Female | Male | Female | | Male | Female | | Male | Female |
| N (%) | 25 (100.00%) | 33 (100.00%) | 15 (100.00%) | 16 (100.00%) | 15 (100.00%) | 16 (100.00%) | | 15 (100.00%) | 19 (100.00%) | | 15 (100.00%) | 15 (100.00%) |
| **SPAN LENGTH** | | | | | | | | | | | | |
| Pc 5 | 5.0 | 4.7 | 5.0 | 5.0 | 4.0 | 5.0 | | 4.0 | 5.0 | | 4.0 | 4.0 |
| Pc 10 | 5.0 | 5.0 | 5.6 | 5.0 | 4.6 | 5.0 | | 4.0 | 5.0 | | 4.6 | 4.0 |
| Pc 20 | 5.0 | 5.0 | 6.0 | 5.0 | 5.0 | 5.0 | | 5.0 | 5.0 | | 5.0 | 4.0 |
| Pc 30 | 5.8 | 5.0 | 6.0 | 5.0 | 5.8 | 5.0 | | 5.0 | 5.0 | | 5.0 | 4.8 |
| Pc 40 | 6.0 | 5.0 | 6.4 | 5.0 | 6.0 | 5.0 | | 5.0 | 6.0 | | 5.4 | 5.0 |
| Pc 50 | 6.0 | 6.0 | 7.0 | 5.5 | 6.0 | 5.0 | | 5.0 | 6.0 | | 6.0 | 5.0 |
| Pc 60 | 6.0 | 6.0 | 7.0 | 6.0 | 6.0 | 6.0 | | 5.6 | 6.0 | | 6.0 | 5.0 |
| Pc 70 | 7.0 | 6.0 | 7.2 | 6.0 | 6.0 | 6.0 | | 6.0 | 7.0 | | 6.0 | 5.0 |
| Pc 80 | 7.0 | 6.2 | 8.0 | 7.0 | 6.8 | 6.60 | | 6.0 | 7.0 | | 6.8 | 5.0 |
| Pc 90 | 8.0 | 7.0 | 8.0 | 7.0 | 7.0 | 8.0 | | 6.8 | 7.0 | | 7.0 | 6.0 |
| **RAW SCORE** | | | | | | | | | | | | |
| Pc 5 | 16.3 | 16.0 | 19.0 | 18.0 | 15.0 | 16.0 | | 16.0 | 17.0 | | 15.0 | 15.0 |
| Pc 10 | 17.6 | 17.4 | 19.0 | 18.0 | 15.6 | 16.7 | | 16.6 | 18.0 | | 15.6 | 15.6 |
| Pc 20 | 18.2 | 18.8 | 21.0 | 18.4 | 17.0 | 18.0 | | 18.0 | 18.0 | | 17.0 | 16.2 |
| Pc 30 | 19.0 | 19.0 | 21.0 | 19.0 | 18.6 | 18.0 | | 18.0 | 19.0 | | 17.80 | 17.8 |
| Pc 40 | 20.0 | 19.6 | 22.0 | 19.8 | 19.4 | 18.8 | | 19.0 | 19.0 | | 18.4 | 18.0 |
| Pc 50 | 21.0 | 20.0 | 23.0 | 20.5 | 20.0 | 19.0 | | 19.0 | 20.0 | | 20.0 | 18.0 |
| Pc 60 | 21.6 | 20.4 | 23.0 | 21.2 | 21.0 | 21.0 | | 20.0 | 21.0 | | 21.0 | 18.6 |
| Pc 70 | 23.0 | 21.0 | 24.0 | 22.0 | 22.0 | 21.0 | | 20.4 | 22.0 | | 22.0 | 19.0 |
| Pc 80 | 23.8 | 22.2 | 24.0 | 23.0 | 22.0 | 21.6 | | 22.0 | 23.0 | | 22.0 | 19.0 |
| Pc 90 | 25.4 | 23.6 | 25.4 | 23.3 | 23.4 | 23.6 | | 22.4 | 23.0 | | 22.4 | 20.0 |
| **PRODUCT SCORE** | | | | | | | | | | | | |
| Pc 5 | 81.5 | 75.2 | 105.0 | 90.0 | 68.0 | 80.0 | | 72.0 | 90.0 | | 60.0 | 60.0 |
| Pc 10 | 88.0 | 87.0 | 110.4 | 90.0 | 72.2 | 83.5 | | 72.0 | 90.0 | | 72.0 | 62.4 |
| Pc 20 | 97.0 | 95.0 | 121.2 | 92.0 | 83.0 | 90.0 | | 81.0 | 95.0 | | 85.0 | 68.8 |
| Pc 30 | 107.4 | 95.0 | 130.8 | 95.0 | 100.6 | 90.0 | | 89.0 | 95.0 | | 89.0 | 78.4 |
| Pc 40 | 116.4 | 100.0 | 138.0 | 99.0 | 116.4 | 94.0 | | 95.0 | 114.0 | | 100.2 | 90.0 |
| Pc 50 | 120.0 | 108.0 | 161.0 | 113.0 | 120.0 | 104.5 | | 95.0 | 120.0 | | 120.0 | 90.0 |
| Pc 60 | 135.6 | 122.4 | 165.2 | 133.2 | 126.0 | 126.0 | | 112.0 | 126.0 | | 129.6 | 93.0 |
| Pc 70 | 149.8 | 130.8 | 182.4 | 138.0 | 132.0 | 131.4 | | 122.4 | 132.0 | | 133.2 | 96.0 |
| Pc 80 | 166.6 | 144.6 | 190.4 | 151.2 | 149.6 | 143.4 | | 132.0 | 161.0 | | 145.2 | 100.0 |
| Pc 90 | 203.2 | 158.2 | 195.2 | 163.1 | 163.8 | 177.6 | | 152.8 | 161.0 | | 154.0 | 114.0 |
| Note. Pc = percentile | | | | | | | | | | | | |

Supplemental Digital Table 3: Percentile distribution for visual processing speed (ms) of the backward corsi tapping task per decade and for males and females separately

| **VISUAL PROCESSING SPEED** | | | | | | | | | | | | | | | | | | | |
| --- | --- | --- | --- | --- | --- | --- | --- | --- | --- | --- | --- | --- | --- | --- | --- | --- | --- | --- | --- |
| Decade | 18-29 | | | 30-39 | | | | 40-49 | | | | 50-59 | | | | 60-69 | | | |
| Sex | Male | Female | | Male | | Female | | Male | | Female | | Male | | Female | | Male | | Female | |
| **SPAN LENTGH 2** | | | | | | | | | | | | | | | | | | | |
| N (%) | 25 (100.00%) | 33 (100.00%) | | 15 (100.00%) | | 16 (100.00%) | | 15 (100.00%) | | 16 (100.00%) | | 15 (100.00%) | | 19 (100.00%) | | 15 (100.00%) | | 14 (93.33%) | |
| Pc 5 | 968 | 864 | | - | | - | | - | | - | | - | | - | | - | | - | |
| Pc 10 | 811 | 794 | | 701 | | 800 | | 1052 | | 1099 | | 1438 | | 923 | | 1316 | | 1138 | |
| Pc 20 | 668 | 682 | | 623 | | 734 | | 906 | | 858 | | 955 | | 885 | | 909 | | 1009 | |
| Pc 30 | 634 | 661 | | 564 | | 694 | | 757 | | 758 | | 813 | | 765 | | 799 | | 981 | |
| Pc 40 | 516 | 572 | | 533 | | 672 | | 692 | | 751 | | 768 | | 689 | | 772 | | 910 | |
| Pc 50 | 507 | 523 | | 524 | | 644 | | 600 | | 709 | | 740 | | 646 | | 746 | | 891 | |
| Pc 60 | 471 | 481 | | 482 | | 618 | | 570 | | 620 | | 696 | | 563 | | 733 | | 857 | |
| Pc 70 | 425 | 456 | | 466 | | 569 | | 550 | | 532 | | 624 | | 547 | | 722 | | 796 | |
| Pc 80 | 351 | 392 | | 452 | | 552 | | 510 | | 525 | | 582 | | 524 | | 615 | | 609 | |
| Pc 90 | 324 | 358 | | 404 | | 432 | | 462 | | 450 | | 528 | | 472 | | 395 | | 480 | |
| **SPAN LENTGH 3** | | | | | | | | | | | | | | | | | | | |
| N (%) | 25 (100.00%) | 33 (100.00%) | | 15 (100.00%) | | 16 (100.00%) | | 15 (100.00%) | | 16 (100.00%) | | 15 (100.00%) | | 19 (100.00%) | | 15 (100.00%) | | 14 (93.33%) | |
| Pc 5 | 1759 | 1605 | | - | | - | | - | | - | | - | | - | | - | | - | |
| Pc 10 | 1429 | 1457 | | 1422 | | 1521 | | 1574 | | 1654 | | 1816 | | 1692 | | 1855 | | 2529 | |
| Pc 20 | 1210 | 1257 | | 1327 | | 1392 | | 1437 | | 1580 | | 1564 | | 1490 | | 1696 | | 1762 | |
| Pc 30 | 1159 | 1156 | | 1279 | | 1302 | | 1389 | | 1478 | | 1485 | | 1469 | | 1631 | | 1657 | |
| Pc 40 | 1113 | 1102 | | 1184 | | 1128 | | 1339 | | 1431 | | 1389 | | 1366 | | 1530 | | 1585 | |
| Pc 50 | 1023 | 1051 | | 1152 | | 1046 | | 1195 | | 1285 | | 1358 | | 1322 | | 1431 | | 1554 | |
| Pc 60 | 948 | 1006 | | 1116 | | 1006 | | 1180 | | 1139 | | 1324 | | 1253 | | 1408 | | 1513 | |
| Pc 70 | 904 | 943 | | 1054 | | 948 | | 1155 | | 1070 | | 1279 | | 1192 | | 1380 | | 1460 | |
| Pc 80 | 829 | 860 | | 1034 | | 934 | | 1111 | | 1031 | | 1173 | | 924 | | 1327 | | 1440 | |
| Pc 90 | 742 | 765 | | 882 | | 896 | | 967 | | 817 | | 1056 | | 851 | | 1174 | | 1187 | |
| **SPAN LENGTH 4** | | | | | | | | | | | | | | | | | | | |
| N (%) | 25 (100.00%) | 33 (100.00%) | | 15 (100.00%) | | 14 (87.5%) | | 15 (100.00%) | | 16 (100.00%) | | 15 (100.00%) | | 19 (100.00%) | | 15 (100.00%) | | 12 (80.00%) | |
| Pc 5 | 2414 | 2471 | | - | | - | | - | | - | | - | | - | | - | | - | |
| Pc 10 | 2117 | 2167 | | 2186 | | 2528 | | 2388 | | 2571 | | 3551 | | 2633 | | 2896 | | 3949 | |
| Pc 20 | 1965 | 1951 | | 2064 | | 2064 | | 2228 | | 2415 | | 2701 | | 2337 | | 2604 | | 3541 | |
| Pc 30 | 1845 | 1848 | | 1855 | | 1903 | | 2134 | | 2139 | | 2601 | | 2253 | | 2402 | | 3186 | |
| Pc 40 | 1734 | 1691 | | 1820 | | 1861 | | 2077 | | 2095 | | 2446 | | 2195 | | 2359 | | 3083 | |
| Pc 50 | 1719 | 1656 | | 1790 | | 1754 | | 2017 | | 2025 | | 2201 | | 2142 | | 2356 | | 2774 | |
| Pc 60 | 1575 | 1571 | | 1748 | | 1724 | | 1914 | | 1945 | | 2100 | | 2069 | | 2204 | | 2506 | |
| Pc 70 | 1475 | 1512 | | 1695 | | 1630 | | 1852 | | 184 | | 1996 | | 1951 | | 2161 | | 2412 | |
| Pc 80 | 1408 | 1468 | | 1663 | | 1560 | | 1776 | | 1680 | | 1933 | | 1671 | | 2134 | | 2043 | |
| Pc 90 | 1308 | 1420 | | 1566 | | 1463 | | 1591 | | 1477 | | 1768 | | 1380 | | 2066 | | 1791 | |
| **SPAN LENTGH 5** | | | | | | | | | | | | | | | | | | |  |
| N (%) | 24 (96.00%) | 31 (93.94%) | 14 (93.33%) | | 11 (68.75%) | | 14 (93.33%) | | 14 (87.50%) | | 13 (86.67%) | | 16 (84.21%) | | 13 (86.67%) | | 6 (40.00%) | |  |
| Pc 5 | 3204 | 4221 | - | | - | | - | | - | | - | | - | | - | | - | |  |
| Pc 10 | 3002 | 3270 | 2584 | | 3698 | | 3114 | | 3885 | | 3834 | | 3512 | | 4134 | | - | |  |
| Pc 20 | 2832 | 2735 | 2471 | | 2955 | | 2858 | | 3273 | | 3429 | | 3465 | | 3581 | | 6220 | |  |
| Pc 30 | 2615 | 2648 | 2440 | | 2700 | | 2820 | | 3139 | | 3170 | | 3223 | | 3307 | | 4967 | |  |
| Pc 40 | 2400 | 2414 | 2295 | | 2527 | | 2650 | | 2870 | | 3098 | | 2934 | | 3229 | | 3767 | |  |
| Pc 50 | 2297 | 2319 | 2266 | | 2415 | | 2522 | | 2793 | | 2990 | | 2800 | | 3145 | | 3423 | |  |
| Pc 60 | 2129 | 2173 | 2235 | | 2316 | | 2454 | | 2523 | | 2953 | | 2735 | | 3111 | | 3262 | |  |
| Pc 70 | 2039 | 2111 | 2195 | | 2221 | | 2390 | | 2419 | | 2904 | | 2647 | | 2970 | | 2704 | |  |
| Pc 80 | 2020 | 1929 | 2129 | | 2097 | | 2338 | | 2318 | | 2670 | | 2495 | | 2903 | | 2512 | |  |
| Pc 90 | 1853 | 1806 | 1951 | | 1931 | | 2303 | | 2156 | | 2253 | | 1966 | | 2797 | | 2435 | |  |
| **SPAN LENTGH 6** | | | | | | | | | | | | | | | | | | |  |
| N (%) | 22 (88.00%) | 26 (78.79%) | 13 (86.67%) | | 10 (62.50%) | | 12 (80.00%) | | 10 (62.50%) | | 8 (53.33%) | | 12 (63.16%) | | 8 (53.33%) | | 3 (20.00%) | |  |
| Pc 5 | 5367 | 5216 | - | | - | | - | | - | | - | | - | | - | | - | |  |
| Pc 10 | 4769 | 4205 | 4026 | | 4095 | | 3582 | | 5191 | | - | | 5322 | | - | | - | |  |
| Pc 20 | 3407 | 3584 | 3424 | | 3308 | | 3478 | | 4568 | | 5155 | | 4427 | | 5825 | | - | |  |
| Pc 30 | 3204 | 3181 | 3189 | | 3127 | | 3297 | | 4010 | | 4193 | | 4005 | | 4415 | | 4849 | |  |
| Pc 40 | 3038 | 3116 | 3067 | | 2930 | | 3188 | | 3302 | | 3792 | | 3634 | | 4007 | | 4269 | |  |
| Pc 50 | 2720 | 2797 | 2726 | | 2615 | | 2967 | | 3125 | | 3670 | | 3514 | | 3973 | | 3689 | |  |
| Pc 60 | 2541 | 2672 | 2674 | | 2547 | | 2741 | | 3013 | | 3515 | | 3417 | | 3805 | | 3299 | |  |
| Pc 70 | 2480 | 2518 | 2629 | | 2425 | | 2657 | | 2823 | | 3291 | | 3275 | | 3387 | | 2909 | |  |
| Pc 80 | 2382 | 2193 | 2514 | | 2319 | | 2525 | | 2491 | | 3179 | | 3179 | | 2866 | | 2714 | |  |
| Pc 90 | 2151 | 2031 | 2089 | | 2086 | | 2390 | | 2334 | | 3173 | | 2954 | | 2684 | | 2714 | |  |
| **SPAN LENGTH 7** | | | | | | | | | | | | | | | | | | |  |
| N (%) | 14 (56.00%) | 19 (57.58%) | 7 (46.67%) | | 4 (25.00%) | | 4 (26.67%) | | 4 (25.00%) | | 5 (33.33%) | | 4 (21.05%) | | 1 (6.67%) | | 2 (13.33%) | |  |
| Pc 5 | - | - | - | | - | | - | | - | | - | | - | | 4499 | | - | |  |
| Pc 10 | 18049 | 4802 | - | | - | | - | | - | | - | | - | | 4499 | | - | |  |
| Pc 20 | 8571 | 4399 | 8360 | | - | | - | | - | | 8654 | | - | | 4499 | | - | |  |
| Pc 30 | 5098 | 4167 | 5861 | | 9373 | | 4932 | | 5764 | | 6728 | | 6074 | | 4499 | | - | |  |
| Pc 40 | 4552 | 3430 | 5206 | | 5764 | | 4794 | | 5236 | | 5618 | | 6052 | | 4499 | | 5630 | |  |
| Pc 50 | 4134 | 3380 | 5062 | | 5593 | | 4547 | | 4859 | | 4917 | | 5653 | | 4499 | | 5061 | |  |
| Pc 60 | 3942 | 3189 | 4470 | | 5422 | | 4300 | | 4482 | | 4848 | | 5254 | | 4499 | | 4491 | |  |
| Pc 70 | 3888 | 3033 | 3832 | | 4543 | | 4079 | | 4000 | | 4720 | | 4216 | | 4499 | | 4112 | |  |
| Pc 80 | 3436 | 2910 | 3334 | | 3664 | | 3857 | | 3518 | | 4481 | | 3178 | | 4499 | | 4112 | |  |
| Pc 90 | 3377 | 2728 | 3076 | | 3664 | | 3857 | | 3518 | | 4401 | | 3178 | | 4499 | | 4112 | |  |
| **SPAN LENTGH 8** | | | | | | | | | | | | | | | | | | |  |
| N (%) | 7 (28.00%) | 9 (27.27%) | 3 (20.00%) | | 1 (6.25%) | | 2 (13.33%) | | 1 (6.25%) | | 2 (13.33%) | | 2 (10.53%) | | 0 (0.00%) | | 1 (6.67%) | |  |
| Pc 5 | - | - | - | | 6274 | | - | | 5819 | | - | | - | | - | | 6395 | |  |
| Pc 10 | - | - | - | | 6274 | | - | | 5819 | | - | | - | | - | | 6395 | |  |
| Pc 20 | 6065 | 4742 | - | | 6274 | | - | | 5819 | | - | | - | | - | | 6395 | |  |
| Pc 30 | 4852 | 4444 | 5466 | | 6274 | | - | | 5819 | | - | | - | | - | | 6395 | |  |
| Pc 40 | 4604 | 3919 | 4935 | | 6274 | | 4598 | | 5819 | | 7071 | | 6635 | | - | | 6395 | |  |
| Pc 50 | 4373 | 3824 | 4403 | | 6274 | | 4390 | | 5819 | | 6415 | | 6490 | | - | | 6395 | |  |
| Pc 60 | 4234 | 3771 | 4284 | | 6274 | | 4182 | | 5819 | | 5759 | | 6345 | | - | | 6395 | |  |
| Pc 70 | 4197 | 3688 | 4165 | | 6274 | | 4043 | | 5819 | | 5321 | | 6248 | | - | | 6395 | |  |
| Pc 80 | 4030 | 3389 | 4105 | | 6274 | | 4043 | | 5819 | | 5321 | | 6248 | | - | | 6395 | |  |
| Pc 90 | 3780 | 2840 | 4105 | | 6274 | | 4043 | | 5819 | | 5321 | | 6248 | | - | | 6395 | |  |
| Note. Pc = percentile; - = not applicable | | | | | | | | | | | | | | | | | | |  |

Supplemental Digital Table 4: Percentile distribution for verbal processing speed (ms) of the letter-number sequencing task per decade and for males and females separately

| **VERBAL PROCESSING SPEED** | | | | | | | | | | | | | | | | | | |
| --- | --- | --- | --- | --- | --- | --- | --- | --- | --- | --- | --- | --- | --- | --- | --- | --- | --- | --- |
| Decade | 18-29 | | | 30-39 | | | | 40-49 | | | | 50-59 | | | | 60-69 | | |
| Sex | Men | Women | | Men | | Women | | Men | | Women | | Men | | Women | | Men | | Women |
| **SPAN LENTGH 2** | | | | | | | | | | | | | | | | | | |
| N (%) | 25 (100.00%) | 33 (100.00%) | | 15 (100.00%) | | 16 (100.00%) | | 15 (100.00%) | | 16 (100.00%) | | 15 (100.00%) | | 19 (100.00%) | | 15 (100.00%) | | 15 (100.00%) |
| Pc 5 | 1291 | 1098 | | - | | - | | - | | - | | - | | - | | - | | - |
| Pc 10 | 1188 | 978 | | 880 | | 1130 | | 1034 | | 1178 | | 1178 | | 1070 | | 1028.00 | | 1420 |
| Pc 20 | 1122 | 914 | | 854 | | 1056 | | 924 | | 1120 | | 922 | | 930 | | 874 | | 984 |
| Pc 30 | 798 | 868 | | 806 | | 928 | | 844 | | 1048 | | 882 | | 860 | | 834 | | 912 |
| Pc 40 | 726 | 830 | | 738 | | 886 | | 800 | | 918 | | 842 | | 860 | | 812 | | 810 |
| Pc 50 | 700 | 810 | | 690 | | 755 | | 720 | | 820 | | 830 | | 830 | | 790 | | 750 |
| Pc 60 | 660 | 772 | | 654 | | 746 | | 632 | | 730 | | 762 | | 780 | | 762 | | 688 |
| Pc 70 | 578 | 694 | | 628 | | 667 | | 616 | | 672 | | 678 | | 750 | | 660 | | 638 |
| Pc 80 | 496 | 568 | | 596 | | 612 | | 592 | | 624 | | 654 | | 700 | | 572 | | 622 |
| Pc 90 | 380 | 450 | | 516 | | 571 | | 504 | | 571 | | 604 | | 490 | | 480 | | 520 |
| **SPAN LENTGH 3** | | | | | | | | | | | | | | | | | | |
| N (%) | 25 (100.00%) | 33 (100.00%) | | 15 (100.00%) | | 16 (100.00%) | | 15 (100.00%) | | 16 (100.00%) | | 15 (100.00%) | | 19 (100.00%) | | 15 (100.00%) | | 15 (100.00%) |
| Pc 5 | 2124 | 2213 | | - | | - | | - | | - | | - | | - | | - | | - |
| Pc 10 | 2014 | 1910 | | 1982 | | 2110 | | 2610 | | 2420 | | 2402 | | 2300 | | 2408 | | 3682 |
| Pc 20 | 1778 | 1760 | | 1746 | | 1964 | | 2190 | | 2248 | | 2144 | | 2180 | | 2122 | | 2808 |
| Pc 30 | 1734 | 1692 | | 1506 | | 1757 | | 1838 | | 2045 | | 1904 | | 1960 | | 1882 | | 2592 |
| Pc 40 | 1684 | 1574 | | 1326 | | 1722 | | 1720 | | 1968 | | 1636 | | 1850 | | 1766 | | 2438 |
| Pc 50 | 1630 | 1480 | | 1270 | | 1495 | | 1560 | | 1890 | | 1590 | | 1730 | | 1720 | | 2290 |
| Pc 60 | 1280 | 1396 | | 1232 | | 1442 | | 1524 | | 1816 | | 1554 | | 1680 | | 1468 | | 2068 |
| Pc 70 | 1120 | 1144 | | 1044 | | 1257 | | 1440 | | 1719 | | 1276 | | 1560 | | 1278 | | 1828 |
| Pc 80 | 1056 | 950 | | 988 | | 1200 | | 1320 | | 1566 | | 1172 | | 1380 | | 1262 | | 1604 |
| Pc 90 | 602 | 862 | | 836 | | 1106 | | 838 | | 1176 | | 994 | | 980 | | 1118 | | 1244 |
| **SPAN LENGTH 4** | | | | | | | | | | | | | | | | | | |
| N (%) | 25 (100.00%) | 33 (100.00%) | | 15 (100.00%) | | 16 (100.00%) | | 15 (100.00%) | | 16 (100.00%) | | 15 (100.00%) | | 19 (100.00%) | | 15 (100.00%) | | 15 (100.00%) |
| Pc 5 | 4785 | 4867 | | - | | - | | - | | - | | - | | - | | - | | - |
| Pc 10 | 4138 | 3796 | | 2792 | | 3587 | | 4278 | | 4420 | | 5312 | | 4580 | | 6328 | | 5656 |
| Pc 20 | 3064 | 3006 | | 2396 | | 2896 | | 3284 | | 3508 | | 3636 | | 3620 | | 5264 | | 4822 |
| Pc 30 | 2872 | 2888 | | 2260 | | 2545 | | 2636 | | 3389 | | 3284 | | 3320 | | 3608 | | 4174 |
| Pc 40 | 2594 | 2754 | | 2082 | | 2362 | | 2436 | | 3178 | | 3148 | | 2590 | | 2968 | | 3656 |
| Pc 50 | 2430 | 2520 | | 1960 | | 2295 | | 2250 | | 3000 | | 3050 | | 2550 | | 2490 | | 3570 |
| Pc 60 | 2208 | 2392 | | 1924 | | 2258 | | 2220 | | 2508 | | 2766 | | 2360 | | 2454 | | 3112 |
| Pc 70 | 2046 | 2134 | | 1814 | | 2241 | | 2050 | | 2451 | | 2490 | | 2320 | | 2086 | | 2990 |
| Pc 80 | 1902 | 1874 | | 1566 | | 2204 | | 1834 | | 2268 | | 1970 | | 1780 | | 1998 | | 2678 |
| Pc 90 | 1460 | 1560 | | 1478 | | 2000 | | 1344 | | 1700 | | 1590 | | 1680 | | 1620 | | 2510 |
| **SPAN LENTGH 5** | | | | | | | | | | | | | | | | | | |
| N (%) | 25 (100.00%) | 32 (96.97%) | 15 (100.00%) | | 16 (100.00%) | | 14 (93.33%) | | 16 (100.00%) | | 13 (86.67%) | | 19 (100.00%) | | 14 (93.33%) | | 11 (73.33%) | |
| Pc 5 | 12939 | 9636 | - | | - | | - | | - | | - | | - | | - | | - | |
| Pc 10 | 7594 | 7067 | 5824 | | 7110 | | 8180 | | 9598 | | 8256 | | 11650 | | 8790 | | 14682 | |
| Pc 20 | 4798 | 4730 | 4068 | | 5994 | | 7400 | | 8170 | | 5856 | | 6200 | | 7680 | | 9726 | |
| Pc 30 | 4264 | 4537 | 3820 | | 5676 | | 6565 | | 5584 | | 4912 | | 5050 | | 5425 | | 7260 | |
| Pc 40 | 4168 | 4236 | 3732 | | 4792 | | 5560 | | 4782 | | 4742 | | 4690 | | 4810 | | 6820 | |
| Pc 50 | 3810 | 3910 | 3630 | | 4095 | | 5155 | | 4550 | | 4300 | | 4530 | | 4055 | | 5760 | |
| Pc 60 | 3566 | 3422 | 3210 | | 3730 | | 3880 | | 4048 | | 3662 | | 4380 | | 3680 | | 5650 | |
| Pc 70 | 3334 | 3227 | 2704 | | 3573 | | 3545 | | 3964 | | 3362 | | 3850 | | 3545 | | 4914 | |
| Pc 80 | 2688 | 2928 | 2440 | | 3130 | | 2400 | | 3548 | | 3242 | | 3180 | | 2930 | | 3732 | |
| Pc 90 | 1824 | 2616 | 2160 | | 2689 | | 2010 | | 2582 | | 2956 | | 2760 | | 2760 | | 3296 | |
| **SPAN LENTGH 6** | | | | | | | | | | | | | | | | | | |
| N (%) | 18 (72.00%) | 18 (54.55%) | 14 (93.33%) | | 9 (52.94%) | | 11 (73.33%) | | 8 (50.00%) | | 6 (40.00%) | | 12 (63.16%) | | 9 (60.00%) | | 2 (13.33%) | |
| Pc 5 | - | - | - | | - | | - | | - | | - | | - | | - | | - | |
| Pc 10 | 10665 | 12077 | 15120 | | - | | 14510 | | - | | - | | 12106 | | - | | - | |
| Pc 20 | 9056 | 9250 | 13980 | | 10430 | | 9690 | | 8758 | | 8854 | | 9608 | | 6790 | | - | |
| Pc 30 | 8095 | 8692 | 7650 | | 9380 | | 8262 | | 8114 | | 6701 | | 8554 | | 6720 | | - | |
| Pc 40 | 7248 | 7482 | 7390 | | 8280 | | 7970 | | 7862 | | 5238 | | 8316 | | 6420 | | 6714 | |
| Pc 50 | 6295 | 6455 | 6480 | | 7760 | | 7900 | | 6930 | | 4505 | | 7715 | | 5090 | | 6015 | |
| Pc 60 | 5808 | 5488 | 5620 | | 7410 | | 5662 | | 5858 | | 4168 | | 7218 | | 5000 | | 5316 | |
| Pc 70 | 5329 | 4797 | 5060 | | 7380 | | 5042 | | 5012 | | 4091 | | 6457 | | 4460 | | 4850 | |
| Pc 80 | 3970 | 4362 | 4010 | | 5960 | | 3644 | | 3936 | | 3822 | | 5458 | | 4280 | | 4850 | |
| Pc 90 | 3410 | 3509 | 3570 | | 5800 | | 3284 | | 3720 | | 3650 | | 5196 | | 4260 | | 4850 | |
| **SPAN LENGTH 7** | | | | | | | | | | | | | | | | | | |
| N (%) | 9 (36.00%) | 6 (18.18%) | 9 (60.00%) | | 4 (25.00%) | | 3 (20.00%) | | 3 (18.75%) | | 1 (6.67%) | | 6 (31.58%) | | 3 (20.00%) | | 0 (0.00%) | |
| Pc 5 | - | - | - | | - | | - | | - | | 7410 | | - | | - | | - | |
| Pc 10 | - | - | - | | - | | - | | - | | 7410 | | - | | - | | - | |
| Pc 20 | 12320 | 15144 | 11690 | | - | | - | | - | | 7410 | | 16332 | | - | | - | |
| Pc 30 | 10730 | 13109 | 10430 | | 14995 | | 8188 | | 8194 | | 7410 | | 11517 | | 9224 | | - | |
| Pc 40 | 10300 | 12052 | 10380 | | 14260 | | 7544 | | 7322 | | 7410 | | 11076 | | 7352 | | - | |
| Pc 50 | 9060 | 10620 | 6760 | | 12045 | | 6900 | | 6450 | | 7410 | | 10385 | | 5480 | | - | |
| Pc 60 | 8490 | 9254 | 6250 | | 9830 | | 5744 | | 5522 | | 7410 | | 9448 | | 4988 | | - | |
| Pc 70 | 7700 | 8428 | 6200 | | 7815 | | 4588 | | 4594 | | 7410 | | 8146 | | 4496 | | - | |
| Pc 80 | 5500 | 6114 | 5950 | | 5800 | | 4010 | | 4130 | | 7410 | | 7432 | | 4250 | | - | |
| Pc 90 | 3990 | 4650 | 5300 | | 5800 | | 4010 | | 4130 | | 7410 | | 7080 | | 4250 | | - | |
| **SPAN LENTGH 8** | | | | | | | | | | | | | | | | | | |
| N (%) | 3 (12.00%) | 0 (0.00%) | 4 (26.67%) | | 0 (0.00%) | | 0 (0.00%) | | 2 (12.50%) | | 1 (6.67%) | | 1 (5.26%) | | 0 (0.00%) | | 0 (0.00%) | |
| Pc 5 | - | - | . | | - | | - | | - | | 14520 | | 11860 | | - | | - | |
| Pc 10 | - | - | . | | - | | - | | - | | 14520 | | 11860 | | - | | - | |
| Pc 20 | - | - | . | | - | | - | | - | | 14520 | | 11860 | | - | | - | |
| Pc 30 | 9678 | - | 14235 | | - | | - | | - | | 14520 | | 11860 | | - | | - | |
| Pc 40 | 7654 | - | 11820 | | - | | - | | 8142 | | 14520 | | 11860 | | - | | - | |
| Pc 50 | 5630 | - | 11325 | | - | | - | | 7335 | | 14520 | | 11860 | | - | | - | |
| Pc 60 | 4642 | - | 10830 | | - | | - | | 6528 | | 14520 | | 11860 | | - | | - | |
| Pc 70 | 3654 | - | 10540 | | - | | - | | 5990 | | 14520 | | 11860 | | - | | - | |
| Pc 80 | 3160 | - | 10250 | | - | | - | | 5990 | | 14520 | | 11860 | | - | | - | |
| Pc 90 | 3160 | - | 10250 | | - | | - | | 5990 | | 14520 | | 11860 | | - | | - | |
| Note. Pc = percentile; - = not applicable | | | | | | | | | | | | | | | | | | |
