# Supplementary figures and images for: Visual and Verbal Working Memory and Processing Speed Across the Adult Lifespan: The Effect of Age, Sex, Educational Level, Awakeness, and Hearing Sensitivity
Source: Front Psychol. 2021 Oct 14;12:668828. doi: 10.3389/fpsyg.2021.668828 (PMC8551836; doi:10.3389/fpsyg.2021.668828)

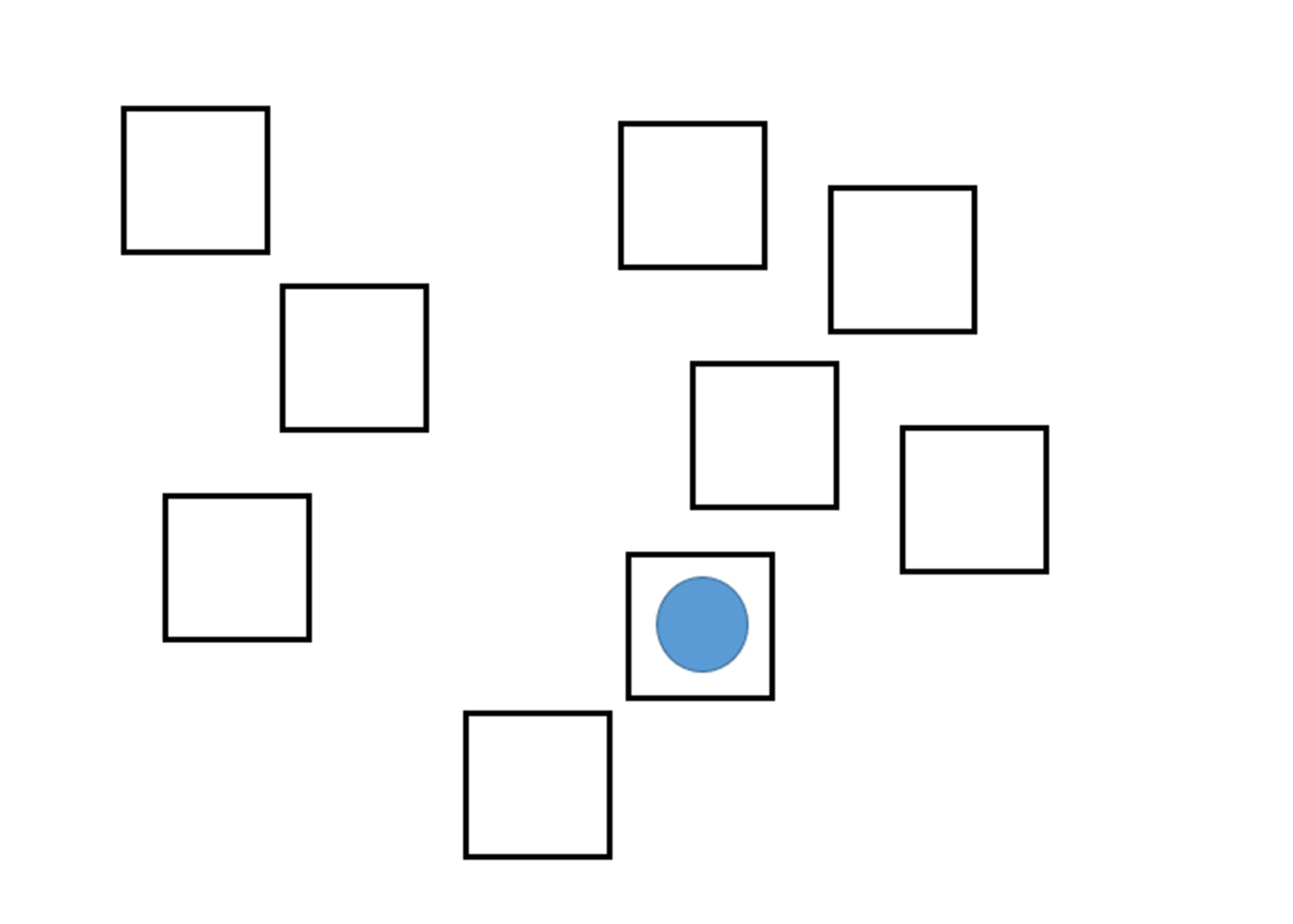

Supplement: Supplementary file 2 [file Image_1.PNG]
